# A new method to identify flanking sequence tags in *chlamydomonas* using 3’-RACE

**DOI:** 10.1186/1746-4811-8-21

**Published:** 2012-06-26

**Authors:** Laurence Meslet-Cladière, Olivier Vallon

**Affiliations:** 1 Centre National de la Recherche Scientifique, Unité Mixte de Recherche 7141/Université Pierre et Marie Curie, Institut de Biologie Physico-Chimique, Paris, 75005, France; 2 Present address : Centre National de la Recherche Scientifique, Unité Mixte de Recherche 7139/Université Pierre et Marie Curie, Station Biologique de Roscoff, Roscoff, 29280, France; 3 Unité Mixte de Recherche 7141/Université Pierre et Marie Curie, Institut de Biologie Physico-Chimique Institut de Biologie Physico-Chimique, 13 rue Pierre et Marie Curie, Paris, 75005, France

**Keywords:** Chlamydomonas, Transformation, Mutant library, 3’ UTR, Antibiotic resistance

## Abstract

**Background:**

The green alga *Chlamydomonas reinhardtii*, although a premier model organism in biology, still lacks extensive insertion mutant libraries with well-identified Flanking Sequence Tags (FSTs). Rapid and efficient methods are needed for FST retrieval.

**Results:**

Here, we present a novel method to identify FSTs in insertional mutants of *Chlamydomonas*. Transformants can be obtained with a resistance cassette lacking a 3’ untranslated region (UTR), suggesting that the RNA that is produced from the resistance marker terminates in the flanking genome when it encounters a cleavage/polyadenylation signal. We have used a robust 3’-RACE method to specifically amplify such chimeric cDNAs. Out of 38 randomly chosen transformants, 27 (71%) yielded valid FSTs, of which 23 could be unambiguously mapped to the genome. Eighteen of the mutants lie within a predicted gene. All but two of the intragenic insertions occur in the sense orientation with respect to transcription, suggesting a bias against situations of convergent transcription. Among the 14 insertion sites tested by genomic PCR, 12 could be confirmed. Among these are insertions in genes coding for *PSBS3* (possibly involved in non-photochemical quenching), the NimA-related protein kinase *CNK2*, the mono-dehydroascorbate reductase *MDAR1*, the phosphoglycerate mutase *PGM5* etc..

**Conclusion:**

We propose that our 3’-RACE FST method can be used to build large scale FST libraries in *Chlamydomonas* and other transformable organisms.

## Background

In advanced genetic models like the green alga *Chlamydomonas reinhardtii*[1], the study of mutants is a powerful tool to elucidate the function of genes. In forward genetics, the genes of interest are *a priori* unknown, and mutants of interest are selected or screened for, based on the alteration of the function under study. In reverse genetics, genes relevant to the function are first identified based on their sequence or genome localization, and the challenge is to obtained mutants in these genes. In both approaches, insertion mutagenesis is the primary method, because it links to the interrupted gene a DNA fragment (usually containing an antibiotic resistance marker) that then serves as a molecular tag of the mutation [2]. The next step is to obtain a piece of DNA that contains the junction between the marker and the host gene, and sequence it. This “Flanking Sequence Tag” (FST) identifies the genomic location of the insertion, its orientation and, if both borders are retrieved, the length of any deletion that may have occurred in the genome. If this approach is to be carried out on a whole genome scale, which is a natural ambition as soon as a model system enters the post-genomic era, a rapid, robust and cheap method is required.

Semi-degenerate PCR is the most popular technique for FST determination. For example in the reference land plant *Arabidopsis thaliana*, Thermal Asymmetric InterLaced PCR (TAIL-PCR) [3] is performed using a marker-specific primer and a degenerate primer that will anneal at random in the genome. This has allowed the development of vast libraries of insertional mutants, with tens of thousands of individual lines now available for public use. In recent years, Ligation-Mediated PCR has replaced TAIL-PCR for *Arabidopsis*[4] and is now applied to other organisms [5]. In *Chlamydomonas*, TAIL-PCR has been used to analyze the first mutant library of significant scale [6], as well as individual mutants (for example, [7]). Other PCR-based techniques relying on degenerate primers, such as RESDA-PCR [8] or SiteFinding-PCR [9] have also been used successfully. Yet, many laboratories, including ours, have found that *Chlamydomonas* DNA is difficult to amplify using degenerate PCR techniques, probably because of the high GC content (65%) of the genome. Ligation-based methods such as GenomeWalker PCR [10] or inverted PCR [11] have also been used. None of these methods is fully reliable and it is often necessary to try several before an FST is obtained. In the context of a large library screen, these techniques are all expected to leave a sizeable fraction of the insertion sites undetermined.

Our long term goal is to generate and store a large library of *Chlamydomonas* insertional mutants with known FSTs. In the present study, we present a new FST identification method that requires neither ligation nor the use of degenerate primers. Using a codon-adapted AadA marker [12] conferring resistance to spectinomycin (Sp), we have generated random insertions in the nuclear genome of *Chlamydomonas reinhardtii*. We have previously reported that the 3’ UTR of RBCS2, present in the original construct, is not necessary for transformation [12]. In transformants obtained with a marker lacking a 3’ UTR, a chimeric mRNA is generated that originates in the marker DNA and terminates in the flanking *Chlamydomonas* genome. We have generated a small collection of mutants. Using oligo-dT primed reverse transcription and nested PCR amplification, we have obtained genome-anchored FSTs from 23 transformants out of 38 randomly selected clones.

## Results

### Transformants obtained with a resistance cassette lacking a 3’ UTR

**Figure 1:**
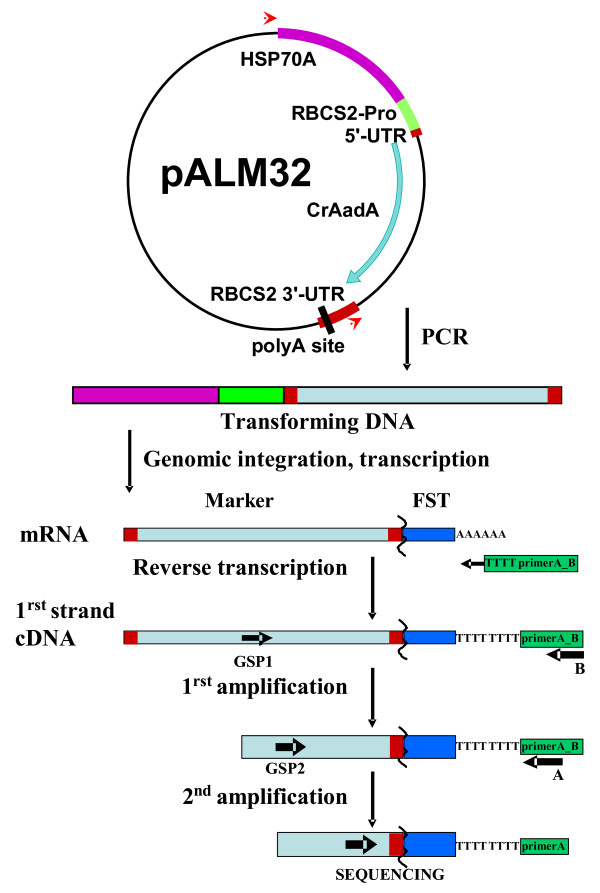
**Principle of FST retrieval by 3’-RACE. A resistance cassette with a truncated 3’UTR is generated by PCR (red arrows).** After its random integration in the genome, transcription from its promoter generates a chimeric mRNA containing the marker (CDS in cyan, UTRs in red) and the flanking *Chlamydomonas* DNA (blue). It is reverse-transcribed using a primer (green) that anneals to its poly-A tail. The first-strand cDNA thus obtained is amplified using a marker-gene specific primer GSP1 and primer B annealing to the end of the extension. A second amplification using nested primers GSP2 and A generates a specific product that is then sequenced using a third marker-specific primer.

In previous experiments, we have found that the CrAadA cassette yields high numbers of transformants when used as a restriction fragment that lacks the 3’ UTR [12]. Here, we used a PCR-amplified fragment that contains the *HSP70A-RBCS2* promoter combination [13] and the recoded AadA CDS but which ends 57 nt after the stop codon, thus lacking most of the *RBCS2* 3’ UTR and the TGTAA signal that direct cleavage and polyadenylation of the mRNA (Figure 1). The purified PCR product was transformed into strain JEX1, either undigested or after digestion with the enzymes *BsiW*I or *SnaB*I (transformations #2, #3 and #4, respectively), for which recognition sites had been introduced by PCR at both ends of the fragment (see Additional file 1). We also studied transformants obtained with the same PCR fragment digested by *Aat*II (transformation #11), which cleaves just before and just after the CDS, thus removing both the 3’ UTR and the promoter. Finally, in order to check the validity of our method in other strains, we also transformed the cell-wall less strain D66 with the uncut PCR product (transformation #14).

After plating on TAP plates containing Sp, we obtained approximatily 50 transformants per electroporation with the cassette lacking the 3’UTR (#2, #3, #4 and #14), compared to only 10 with the CDS alone (#11) and about 100 with a control PCR product encompassing the full 3’ UTR. For a given fragment, we saw no significant effect of the restriction enzyme digestion of the ends of the cassette on the transformation yield (data not shown). This shows that the nature of the DNA ends (recessed or blunt, phosphorylated or not) has little impact on transformation efficiency. For our FST-retrieval attempts, we randomly picked 38 transformants, ten from each of the transformations #2, #3 and #4, and four from transformations #11 and #14. All of the transformants were resistant to 200 μg/ml Sp initially, even if 3 eventually lost antibiotic resistance partially or completely after a year of propagation in the absence of the antibiotic. None of the transformants had obvious color or growth defects on TAP or Minimum medium.

### FST sequencing

**Figure 2:**
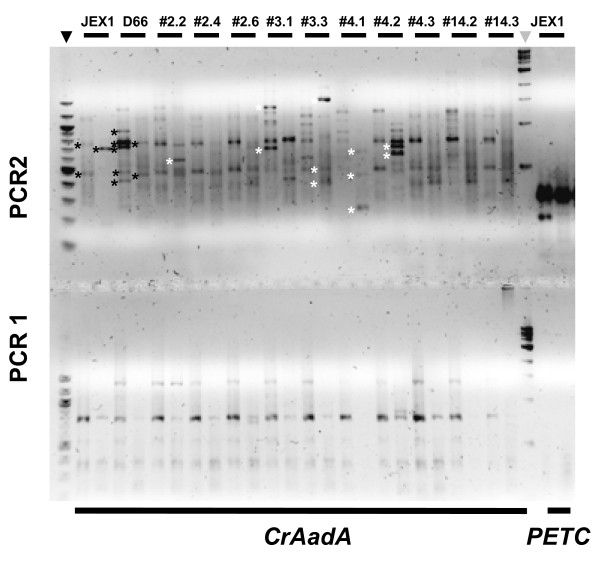
**3’-RACE amplification of flanking DNA. Strains are indicated at the top. Regular Phusion polymerase was used to amplify the CrAadA marker (left) or the control PETC transcript (right).** Bottom panel: PCR1; top: PCR2. The New England Biolabs size ladders are indicated by arrowheads (black: 100 bp ladder; grey: 1 kb ladder). Black stars point to background bands found in negative controls, white stars to mutant–specific bands that eventually yielded FSTs. For each strain, the left lane shows samples where PCR used annealing at 60 °C, while the right lane used « touch down » from 72 to 60 °C in 12 cycles. Note that the latter yields less background and in general stronger specific bands.

The resistance of the transformants to Sp suggests that a chimeric mRNA is formed that starts in the resistance marker and ends in the flanking DNA. Therefore, we prepared total RNA from transformants grown in liquid TAP in presence of antibiotic and performed RT-PCR using primer Q_T_ (Figure 1). This primer anneals to the poly-A tail, yielding a first strand cDNA with a 35 nt 5’ extension upstream of the oligo-dT. We then amplified the AadA cDNA using the marker-specific forward primer CrAadA_F1, and primer Q_O_ which anneals to the first 18 nt of the 5’-extension provided by Q_T_. This first amplification (PCR1) usually resulted in only faint non-specific PCR products, similar in transformants and control strains (Figure 2). In a second, nested amplification (PCR2), we used a forward primer annealing further downstream in the marker,(CrAadA_F2) along with the reverse primer Q_I_ annealing to the last 18 nt of the Q_T_ extension, downstream of Q_o_. This time, we obtained PCR products of different sizes (Figure 2, top panel) suggesting that they arose from specific amplification of chimeric mRNAs, which we subsequently confirmed (see below). As a control, we also analyzed 3 transformants obtained with a longer PCR fragment that contained the entire RBCS2 3’ UTR (see Additional file 1, p. 1) and found that the expected product was already visible after the first amplification. Sequencing of the 360 nt PCR2 product revealed the expected fusion between the CrAadA marker and the downstream RBCS2 3’ UTR (Additional file 1). The yield of this product was not increased by a 30 min 40 °C heat shock (not shown), therefore we did not include this step in our routine protocol for analysis of transformants. Still, enhanced transcription after heat shock [13] could be beneficial for obtaining some products that are difficult to amplify. As a control for the quality of our RNA preparation and PCR method, we verified that all control and transformant samples produced strong amplification at PCR2 when the CrAadA primers were replaced by primers annealing to the 3’ UTR of the highly expressed endogenous *PETC* gene (Figure 2). The CrAadA amplification gave weaker bands, suggesting an overall lower level of expression.

**Figure 3:**
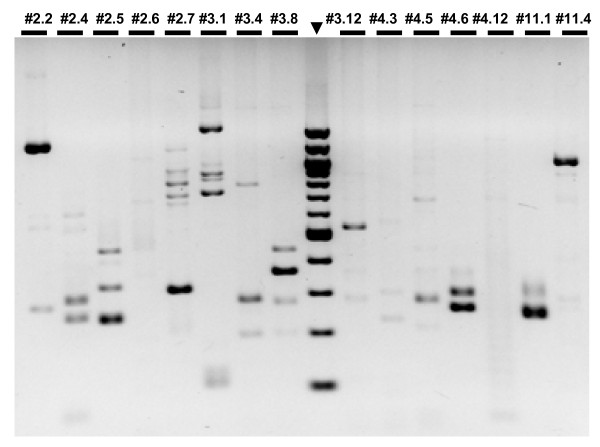
**3’-RACE amplification of flanking DNA (PCR2). Strains are indicated at the top.** Hot Start Phusion polymerase and primer set 2 were used in a touch-down PCR reaction. The 100 bp ladder is indicated by an arrowhead.

As we were trying to establish a method of general applicability, we did not try to optimize the PCR conditions for every strain, but rather aimed to find conditions that would yield the highest amount of information from the group of transformants under study. We found that touch-down PCR for both the initial and the nested PCR improved the yield of mutant-specific bands (Figure 2), and that the use of HotStart Phusion polymerase gave more reproducible results than regular Phusion (compare Figure 3 and Figure 2). Initially, we carried out a third round of amplification using Q_I_ and another marker-specific primer, CrAadA_F3, but we found that this in general did not improve the quality of the FST. Sequencing was done commercially using the Sanger dideoxynucleotide method. In our first experiments, we endeavored to separate the PCR2 or PCR3 products on agarose gels and the extracted DNA was sent for sequencing. However, interpretable results could also be obtained when the PCR product was sent directly for sequencing, even when multiple bands were observed on agarose gels (see below). In this case, it was sometimes necessary to analyze the chromatograms individually to disentangle overlapping sequences. At a late stage in the development of the method, we used for the 3’ end a different set of primers, mimicking the Illumina adapter (Q_S_, Q_U_ and Q_D_, set 2, see Additional file 1), in combination with the same marker-specific primers (Figure 3). This slightly improved the efficiency of FST determination for strains that had initially failed, and we now recommend this set as more robust.

Table 1 and Additional file 1 describe the FSTs obtained, from a total of 10 experiments grouping 3 to 22 transformants each. Each transformant was subjected to 1, 2, or a maximum of 3 sequencing attempts. In the end, we were able to obtain 28 valid FSTs from 27 of our 38 transformants (71%). A sequence was considered as a valid FST when sequence continuity could be established between the cassette and more than 30 nt of surrounding *Chlamydomonas* DNA (NB: continuity was not required for transformation #11 where the *Aat*II restriction site used is so close to the sequencing primer that we do not expect to be able to read the junction). Sequences were considered Failures when there were : absence of specific bands; sequence of low quality or lacking a recognizable marker portion; very short FSTs caused by premature polyadenylation; or PCR contamination from the control transformants carrying the full RBCS2 3’ UTR. Some strains gave rise to multiple poly-A tracts, or to multiple bands yielding staggered FSTs pointing to the same genomic location, indicating that polyadenylation happened at multiple sites along the chimeric transcript. Transformant #2.1 yielded two completely different FSTs, suggesting two independent insertions. In addition, several valid FSTs could not be mapped unambiguously to the *Chlamydomonas* genome, the most common reason being that they fell in a repeated sequence. The FST retrieved from transformant #2.6 was composed of two transposable element fragments (DNA-2-7_CR, followed by TOC1), a combination that is not found as such in the reference genome sequence. This suggests that either insertion of the marker activated a transposition event, or that the insertion locus lies in one of the sequence gaps of the genome. For transformant #4.2, the FST could not be mapped onto version 4 of the genome, but it could be mapped to a partially finished version 5, kindly communicated to us by Jane Grimwood. In total, 23 FSTs could be mapped to a unique location in the genome, while 5 remained of uncertain location. Among the mappable FTSs, four fell in intergenic regions, based on annotation of transcripts (Augustus 10.2). Of insertions into genes, one fell in a 5’ UTR exon, five in 3’ UTRs, three in coding exons and ten in introns.

**Table 1:** Summary of FST determination. Lines in italics indicate absence of valid FST

**trans- formant**	**# of trials**	**valid FSTs**	**chromosome**	**FST position**	**locus**	**annotation**	**type of location**	**orientation/ gene**	**marker resection (nt)**	**notes**	**confirmation by genomic PCR**
**#2.1**	3	2	16	304054-303727	Cre16.g649785/Cre16.g649752	no predicted function/ no predicted function	intergenic	not applicable	9		unsure, PCR failed
		2	19	810984-811036	Cre19.g757350	NAC domain protein	3' UTR	+	8	A 37 nt fragment of the PCR product is inserted between the active cassette and the flanking DNA; poly-A site not recorded before for the host gene	unsure, PCR failed
**#2.2**	2	1	1	8429714-8432270 (spliced)	Cre01.g060850	PSBS3, Chloroplast PSII-associated 22 kDa protein	exon 3; FST extends up to exon 10, with splicing	+	0	expression of interrupted gene is not documented by 454 or Illumina data	confirmed
**#2.3**	2	1	12	589974-589850	Cre12.g488050	FFT5, Fructan fructosyltransferase	intron 2	-	0		confirmed
**#2.4**	1	1	14	3326333-3326254	Cre14.g630200	no predicted function	3' UTR	+	0	not the endogenous poly-A site; part of the FST is hidden below an early poly-A tail	unsure, PCR failed
**#2.5**	3	2	12	3269654-3269776 and divergent 3268980-3269003	Cre12.g512250	protein with HRDC domain	intron 4	+	0	two staggered polyadenylation sites	not tested
**#2.6**	1	1	?	?	?	transposable elements TOC1 and DNA-2-7_CR	not applicable	not applicable	0	UNMAPPED: could be in an unsequenced region, or due to a rearrangement	not tested
***#2.7***	*2*	*0*	*-*							*FAILED: amplifies a sequence from Cre10.g429850 exon 7 but the junction with the cassette cannot be read*	*not tested*
**#2.8**	1	1	27	75383-75179	Cre27.g774700	SGNH hydrolase	5'-UTR	+	?	poly-A tail at end of cassette masks junction with flanking DNA	confirmed
**#2.9**	1	1	9	2341565-2341083	Cre09.g400950	Major Facilitator Superfamilly	3’ UTR	+	3	uses endogenous poly-A site	confirmed
**#2.11**	2	1	17	1621018-1620917	Cre17.g707950/Cre17.g708000	HEP1 escort protein/ PAS domain protein	intergenic	not applicable	0	there are several other good matches, but this is the only perfect one	confirmed
**#3.1**	3	0								*FAILED: no marker DNA in sequence*	-
**#3.2**	2	3	12	9166015-9166885	Cre12.g560350	CNK2, NimA-related protein kinase 2	intron 1 (splits 5'-UTR)	-	0 (filled in)	at least two staggered polyadenylation sites	confirmed by Lynn Quarmby (pers. comm.)
**#3.3**	1	2	7	1015855:1015936 and 1015996-1016192	Cre07.g319550	FIST C domain protein	intron 1	+	0 (filled in)	two staggered polyadenylation sites	confirmed
***#3.4***	*3*	*0*							*0 (filled in)*	*FAILED : FST too short (1 nt)*	*-*
**#3.5**	1	1	?	Chr_7:2564723–2565098 and other locations	genes similar to Cre07.g332350	unknown function	usually intron 1	+	9 (incl. overhang)	UNMAPPED: maps equally well in several homologous genes	-
**#3.6**	2	1	3	2440121-2440156	Cre03.g166950	PGM5, phosphoglycerate mutase	intron 6	+	0 (filled in) + additional G		confirmed
***#3.7***	*2*	*0*								*FAILED: amplifies a sequence from Cre14.g632700 exon 20 but the junction with the cassette cannot be read*	*disproved (gene intact)*
**#3.8**	3	1	?	?	?				0 (uncut)	UNMAPPED: the 35 nt FST maps to several locations	-
**#3.11**	2	2	17	2083314-2083570	Cre17.g712100	MDAR1	intron 7	+	0 (filled in)	one FST suggests artifactual splicing between end of marker and exon 8	unsure, PCR failed
**#3.12**	2	1	10	1630598-1630551	Cre10.g429850	protein of unknown function conserved in Chlorophyceae	intron 6	+	0 (uncut)	2 insertions ? underneath the main sequence, you can also read a short FST corresponding to a repeated sequence	unsure, PCR failed
**#4.1**	1	2	5	377208-377154 and −377020	Cre05.g231500	Zn-finger protein	intron 6	+	0 (uncut)	two staggered polyadenylation sites	confirmed
**#4.2**	1	1	8	v5:4490830-4491010	Augustus_ 11.2|g9033.t1	unknown function	3' UTR	+	0 (uncut)	a good FST, not found in version 4 genome but found in three unpublished genome assemblies	not tested
***#4.3***	*2*	*0*								*FAILED: no good sequence*	*-*
**#4.4**	2	1	2	9598215-9596562	Cre02.g115000	Ribosome-binding factor A	intron 4	+	0 (uncut)	two staggered polyadenylation sites; intron 5 is retained in the chimeric mRNA, but intron 6 is spliced out	confirmed
***#4.5***	*3*	*0*								*FAILED: no good sequence*	*-*
**#4.6**	2	2	?	?	?					UNMAPPED: a 35 nt FST mapping to several locations, and a long one not mapping at all	-
**#4.8**	1	2	7	698506-698423 and −698252	Cre07.g317300/ Cre07.g317350	MAPKKKK1 and a protein of unknown function	intergenic	not applicable	0 (uncut)		confirmed
***#4.9***	*1*	*0*								*FAILED: polyadenylation starts within the marker*	*-*
**#4.10**	1	1	7	4591244-4590811	Cre07.g346000	unknown function	end of 3'UTR	+		poly-A site downstream of gene model	confirmed
***#4.12***	*1*	*0*								*FAILED: no good sequence*	
**#11.1**		1	16	2391524-2391584	Cre16.g666300	protein kinase	upstream, intergenic	not applicable	?	the PCR product cannot be read in the FST (primer too close to end)	disproved (gene intact)
***#11.3***	*1*	*0*								*FAILED: no marker DNA in sequence*	*-*
**#11.4**	1	1	4	704462-705281	Cre04.g215800	no annotation	last exon	+	2		unsure, PCR failed
***#11.5***	*1*	*0*								*FAILED: no match to genome*	*-*
**#14.1**	3	1	14	2909515-2909394 and 2908783- 2908718	Cre14g.627600	Dynein heavy chain	intron 6 and exon 8	+	0	evidence for genome rearrangement or aberrant splicing	disproved (gene intact)
**#14.2**	1	2	12 and other	> a dozen locations (with splicing)	many	Chlamydomonas- specific kinase family	usually exon 4	+	0	UNMAPPED: too many good hits	-
**#14.3**	3	1	14	3326333-3326254	Cre14.g630150/ Cre14.g630200	TRAF-type zinc finger protein; protein of unknown function conserved in Volvox only	intergenic	not applicable	0		unsure, PCR failed
***#14.4***	*3*	*0*								*FAILED: FST too short (2 nt)*	*-*

### Verification by genomic PCR

**Figure 4:**
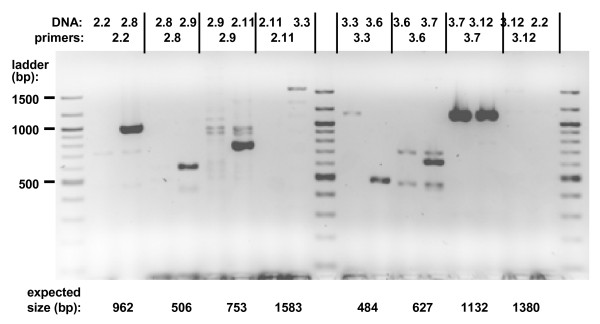
**Verification of selected insertions using genomic PCR (HotStart Phusion).** The DNA and primer pair used in each amplification are indicated at the top of the figure. The 100 bp ladder is loaded in the center and outermost lanes; the size of some of its bands is indicated on the left.

In order to independently verify that we had indeed determined the correct insertion site, we isolated crude genomic DNA from 19 of the transformants giving FSTs and subjected them to PCR, using primers designed to amplify the gene across the insertion point (Figure 4). The quality of each primer pair and each DNA preparation were checked using a positive control combination of strain and primer pair. Out of 13 tests where the expected control amplifications were obtained, we verified in 11 cases that the gene was indeed disrupted in the transformant. For two transformants, we found that an intact copy of the gene was still present in the genome, suggesting that the FST was an artifact, or that the gene underwent duplication concomitant with cassette integration. One additional transformant that we did not check (#3.2) was independently verified as carrying an insertion at the expected position (Lynne Quarmby, University of British Columbia, personal communication).

## Discussion

### An efficient method for FST retrieval

The goal of this study was to devise a simple and reliable method for identification of FSTs in *Chlamydomonas*. We took advantage of the serendipitous observation that transformants can be obtained using a selection marker carrying a truncated 3’ UTR [12]. This suggested to us that antibiotic resistance could rely on the production of a chimeric mRNA, starting in the marker and reading into the insertion flank where cleavage and polyadenylation of the pre-mRNA would occur. Here, we have amplified such chimeric mRNAs using a simple 3’-RACE protocol [14], with only minor modifications. This allowed us to retrieve valid FSTs from 71% of the transformants we examined. Almost all of these FSTs were uniquely mappable onto the genome. In 11 of 13 cases tested, the identification of the insertion locus was corroborated by genomic PCR, i.e. no amplification was obtained in the transformant with primers flanking the insertion point. In two cases, we were unable to observe the gene interruption predicted by the FST when we analyzed the DNA of the transformants: either the FST was an artifact, or a local duplication occurred at the site of the insertion. Overall, our method compares well, in terms of ease, success rate and reliability, with other published methods. It is also quite versatile, and can be adapted to markers other than CrAadA. Recently, our method has been used with an AphVIII-containing paromomycin resistance cassette, with very satisfactory results (Jae-Hyok Lee, University of British Columbia, personal communication).

We emphasize that we intentionally limited our efforts to retrieve any particular FST, because we wanted to test the validity of the method in a medium-throughput mode. In many cases, the PCR product was submitted to Sanger sequencing without purification of the specific products. We believe that the success rate and robustness of the method can be improved, for example by exploring more RT-PCR primers or by systematically reamplifying DNA fragments when several bands are found, as suggested in [8]. However, as it stands, our 3’-RACE FST method still represents a valuable addition to the molecular biology toolkit of *Chlamydomonas*. Its major advantage is that it does not use degenerate primers that can make methods such as TAIL- or RESDA-PCR difficult to perform in *Chlamydomonas* (our unpublished results). No enzyme digestion or adapter ligation is required, in contrast to GenomeWalker and Adapter-Ligation Mediated PCR, and the ability to retrieve an FST does not depend on the vicinity of any particular restriction site. Also, RNA is more abundant in the cell than DNA, allowing the use of small culture volumes. Our 20 ml cultures yielded RNA sufficient for hundreds of reactions, so culture volumes in the ml range could be considered.

In its essence, our method is symmetrical to the promoter-trap experiments that have been carried out in a number of organisms including *Chlamydomonas*[15], where a promoterless construct randomly introduced into the genome relies on an endogenous promoter for its expression. In comparison, our 3’-RACE FST method can hit a wider diversity of sites, since all that is required is the presence downstream of the introduced cassette of some sequence recognizable by the PolII polymerase complex as a cleavage and polyadenylation signal, even with low efficiency. In *Chlamydomonas*, the main signal has been identified as TGTAA, located on average 14 nt upstream of the polyadenylation site [16,17]. This simple sequence motif occurs on average every 1.5 kb in *Chlamydomonas*, but because some degeneracy is allowed, it is expected that a large fraction of the insertions would fall not too far upstream of such a sequence, allowing expression of the gene at a level sufficient for antibiotic resistance. Clearly, a TGTAA sequence is not strictly necessary to determine termination of the transcript. Only 12 of the 26 poly-A tails we recorded showed a TGTAA sequence within 22 nt upstream of the cleavage point, and while many of the others presented similar sequences fitting one of the variations observed by [17], seven did not. The native polyadenylation site of the host gene was observed in only one case. The observed degeneracy of polyadenylation signals is in line with the finding that there was no bias in favor of insertion in the 3’-UTR of genes (only 5 out of 18 mappable FSTs), as could have been feared.

Like any method, ours has its limitations. One of them is that the 3’-UTR less cassette transforms with a lower efficiency compared with a complete cassette (approximately half, see also Figure 1B in [12]). But this is a moderate effect, compared to the 5-fold reduction observed when the promoter region is omitted. By and large, it results in the generation of a sufficient number of transformants for any type of library. Indeed, most of the mutants isolated by the Grossman laboratory using their strategy of target-specific genomic PCR of pooled mutants [18] were obtained with a cassette carrying a truncated 3’ UTR, the stated aim being to reduce the distance between the marker-specific primer in the CDS and the target gene. These authors also noted the possibility that chimeric mRNAs can be formed, but they rather considered it as a drawback.

A more serious limitation is that that the chimeric mRNA may be of low abundance and may contain multiple weak polyadenylation sites, thus yielding no or many faint PCR products. The sequences that we have obtained indeed vary a lot in length and quality (only the best ones for each transformant are shown in Additional file 1). With such small amounts of RNA to amplify, contamination by background bands and by DNA from other PCR reactions can also prove a severe limitation. But if high-throughput sequencing is put to use in the context of a large insertion library, then we can hope to detect even low abundance transcripts. Another limitation is that the cleavage and polyadenylation signal can occur by chance shortly after the insertion site, yielding an FST too short for mapping. Usually, however, it is expected that longer transcripts will also be produced, which could yield FSTs of sufficient length. Still, early polyadenylation has led to failure in two cases in this study. In fact, we eventually realized that the PCR product which we used was far from optimal, since it contained a TGTAA sequence 25 nt before its end. This sequence was contributed by the end of the *RBCS2* CDS, where it does not function as a cleavage and polyadenylation signal. We did not seek to remove it, which in retrospect was clearly a mistake. This is another area where our protocol can easily be improved.

### Preferred insertion in transcribed regions

When we examined the site of insertion of the cassette in our transformants, we realize that genes were preferentially hit, compared to intergenic regions (see Table 1). While genes are predicted to occupy 54% of the *Chlamydomonas* genome [19], 23 of our 28 FSTs lie within genes versus 5 in intergenic regions and transposable elements. These numbers suggests that the cassette is better expressed when it inserts into transcribed regions. Among the genes that we identified in our mini-collection, several are worth noticing. *PSBS3* (interrupted in transformant #2.2) is one of three homologues of the plant PsbS gene, which has been shown to be involved in non-photochemical quenching of photosynthetic light excitation [20]. *RBF1*, the ribosome factor A, is also a chloroplast-targeted protein possibly involved in rRNA biogenesis. The monodehydroascorbate reductase *MDAR1* is probably part of the antioxidant defense system [21], while *CNK2* has been shown to regulate flagellar length and cell size in *Chlamydomonas*[22]. Other affected genes have been proposed putative functions (a glycoside hydrolase belonging to family 32; the phosphoglycerate mutase *PGM5*; a putative transporter of the Major Facilitator Superfamilly). Others contain domains suggesting various molecular functions (protein kinase, FIST C, PAS, Zinc-finger, NAC, HRDC, SGNH hydrolase).

Interestingly, we observed a strong bias in the orientation of the cassette with respect to the interrupted gene. In 20 cases, the transcription of the cassette used the same strand as the host gene, and in only two cases was it in the opposite direction. We think that this is not due to a bias in our ability to retrieve FSTs by 3’-RACE, as Gonzalez-Ballester and co-workers, using a DNA-based method, also found an excess of sense vs. antisense orientation (31 vs. 15) [18]. Rather, we propose that a cassette inserting in opposite orientation within a transcribed gene would be subject to anti-sense effects, lowering its expression level below the resistance threshold. To improve transformant yield, it might be a good idea in the future to include inward-reading cleavage/polyadenylation signals at both ends of the cassette. This would reduce the chance that transcripts from surrounding promoters read into the cassette and cause it to become silenced. We note that even if the cassette is in the sense orientation, expression of the interrupted gene is likely to be severely disrupted. Insertions lying within introns are also expected to prevent gene expression, because *Chlamydomonas* uses an intron definition model to splice its usually short introns [23].

### Modification of the DNA ends upon transformation

It is generally assumed that transformation involves the formation of a double stranded break in the genome and its repair by non-homologous end-joining [24]. The key event is the association of the Ku70-Ku80 complex, both with the free ends of the genomic DNA and with the ends of the transforming DNA. Ku binds with high affinity to all sorts of DNA ends, and we were therefore not surprised that similar transformant yields were obtained with restriction-digested or uncut PCR products. As part of the end-joining process, the DNA ends can be modified, and we indeed observed evidence for such processes. For example, the *BsiW*I overhang was usually filled in, consistent with repair by a polymerase. In a few cases (#2.1, #3.6), untemplated nucleotides have clearly been added between the marker and the FST. Resection of the marker DNA was a concern for FST retrieval, and we were thus relieved to see that only 4 of our insertions (out of 24 scored) showed evidence for marker resection. Maximum resection was 9 nt i.e. far less than would be necessary to destroy the primer binding site. Another concern if one wants to correlate the FST with a phenotype is that the genome should not carry large deletions that would affect genes on the other side of the marker. We have not tried to sequence the other flanking sequence of our insertions, but Gonzalez-Ballester and co-workers have found that in general their cassette lacking the 3’UTR yielded no or only short genomic deletions [18].

In our experiments, we observed cases where the cassette was ligated to another copy of the transforming DNA or a fragment (see for example transformants #2.1) at the site of insertion into the genome. Indeed, tandem cassettes insertions are quite frequent in Chlamydomonas transformation. In early experiments, we also found that carrier salmon DNA used in our initial electroporation protocol could be inserted together with the marker and thus form the FST. In experiments with digested plasmids, we have also observed that religation could involve the sticky ends generated by the restriction enzyme used (data not shown). The presence of microhomology at the end of the joined molecules is known to greatly favor end-joining [24]. All these observations lead us to recommend the use of purified PCR product, at the lowest possible concentration and with no modification or cleavage of the DNA ends.

## Conclusion

We describe a new method for FST retrieval by 3’ RACE in *Chlamydomonas* and show that it can be used with a reasonable success rate on a small number of transformants. Because any transformant obtained with a cassette lacking the 3’UTR can also be subjected to DNA-based methods for FST retrieval, as was done in ref [18], we would advise any mutant-library generation project to start with such a truncated cassette, so that both the 3’-RACE and the DNA-based methods can be used to retrieve the desired FSTs. We believe that the main limitations of our method, i.e. the overall low abundance of the chimeric mRNA and the multiplicity of weak polyadenylation sites, can be easily overcome if the power of high throughput sequencing is brought to bear. We are currently adapting our 3’-RACE FST protocol for the Illumina sequencing of a large number of mutants in parallel. We also hope that this method will be applicable not only to *Chlamydomonas*, but also to other organisms where it is desired to retrieve FSTs from libraries of insertional or transposon-tagged mutants.

## Materials and methods

### Strains

Two strains of *Chlamydomonas reinhardtii* have been used in this study. Strain D66 (cw15, nit2, mt+) [25] was kindly provided by S. Lemaire and is used in many insertion libraries [18]. The walled strain JEX1 (nit1 nit2 mt-) is a 137c derivative generated by X. Johnson in our laboratory [26] and screened as highly efficient in electroporation transformation. Strains were grown at 23 °C under continuous low light on TAP [1].

### Transformation of *C. Reinhardtii*

The CrAadA cassette lacking the 3’ UTR was amplified by PCR from pALM32 [12] with primers 70A_Up and RBCS_Dw (see primers in Additional file 2). For the control transformation (with 3’ UTR), RBCS_Dw was replaced by BS_KpnDw. Nuclear transformation was performed with 1 μg DNA by electroporation essentially as in [12], except that the walled strain JEX1 was electroporated at 1 kV. Carrier DNA was omitted and digestion mixtures were used without purification. Plating was done without starch on TAP plates containing 75 to 200 μm/ml Sp.

### Amplification of FSTs by 3’-RACE

Total RNA was isolated using hot-phenol extraction [27], from 20 ml cultures in 50 ml Falcon tubes on a rotating wheel. Reverse transcription used the SuperScript III First-Strand kit from Invitrogen in a DNA Engine PCR machine (MJ research). Primer Q_T_ was mixed with 5 μg RNA, incubated at 65 °C for 5 min then cooled to 55 °C over 100 sec, after which the annealed mixture was kept at 0 °C. The RT-mix was added and incubation proceeded at 25 °C for 5 min, then 50 °C for 50 min. After inactivation at 85 °C (5 min) and cooling to 0 °C, RNAseH was added and left to degrade template RNA for 20 min at 37 °C. The resulting cDNA was diluted 20-fold in TE buffer and kept at 4 °C.

3’-RACE was performed as described in [14]. Touch-down PCR1 used primers Q_O_ and CrAadA_F1. As a template, 0.2 μl of a 200-fold dilution of the cDNA was used in a 10 μl reaction, using the Phusion enzyme (Finnzyme) in GC-buffer. After 5 min denaturation at 98 °C, hybridization was carried out for 15 sec at progressively decreasing temperature (from 72 °C to 60 °C over 12 cycles), then elongation proceeded for 3 min at 72 °C. The following 22 cycles used a constant annealing temperature of 60 °C, and the final elongation step was extended to 15 min. PCR2 was carried out in a 40 μl volume using the same program, with primers Q_I_ and CrAadA_F2, and as a template 0.2 μl of a 200-fold dilution of the PCR1 product. Alternatively, primers Q_T_, Q_O_ and Q_I_ were replaced by Q_S_, Q_U_ and Q_D_ (see Additional file 2). Sanger sequencing of PCR products or bands cut-out from agarose gels (purified using the PCR purification and QIAEX II kits from Qiagen, respectively), was performed by Eurofins (Ebersberg, Germany) using primer CrAadA_F4. Sequences were mapped using BLAST [28] to the Chlamydomonas v4 genome (http://www.phytozome.net) and its Augustus 10.2 annotation

## Abbreviations

FST, Flanking sequence tag; RACE, Random amplification of cDNA ends; Sp, Spectinomycin; TAIL-PCR, Thermal Asymmetric InterLaced PCR; UTR, Untranslated region.

## Author’s contributions

LMC and OV both performed the experiments and wrote the manuscript. All authors read and approved the final manuscript

## Supplementary Material

Additional file 1 Sequence of PCR products and FSTs obtained. Click here for file

Additional file 2 Additional table : primers used in this study. Click here for file
