# Supplementary material for: A new method to identify flanking sequence tags in chlamydomonas using 3’-RACE
Source: Plant Methods. 2012 Jun 26;8:21. doi: 10.1186/1746-4811-8-21 (PMC3439250; doi:10.1186/1746-4811-8-21)
Supplement: Additional file 1 — Sequence of PCR products and FSTs obtained. [file 1746-4811-8-21-S1.pdf]

A new method to identify Flanking Sequence Tags by 3'-RACE

Laurence Meslet-Cladiere [1] and Olivier Vallon [*]

Supplemental material

I) Oligonucleotides used to generate the PCR products

The part annealing to the pALM32 template is highlighted in green
The BsiWI (C^GTACG) site is underlined, the **SnaBI** site (TAC^GTA) is in bold

```
>70A_Up
GTCCATACGTACGACCCTCACTAAAGGGAACAAAAGCTGGAG

>RBCS_Dw
GTGTCTACGTACGCCTCCATTTACACGGAGC

>BS_KpnDw
GTAGCTACGTACGACTCACTATAGGGCGAATTGGG
```

Primers 70A_Up and RBCS_Dw were used for transformations #2, #3, #4 and #14, primers 70A_Up and BS_KpnDw for the control transformation with a 3'-UTR

II) Sequence of the PCR product for transformations #2, #3, #4, #11 and #14.

The HSP70A promoter region is highlighted in pink, the RBCS2 promoter in red, its 5'-UTR in yellow; the CrAadA CDS is in blue, with the region coding for the cTP underlined. The marker-specific primers used for 3'-RACE are in red, the sequencing primer is underlined. The sites for AatII, used for transformation #11, are highlighted in grey

```
>PCR_2 (2248 nt)
GTCCATACGTACGACCCTCACTAAAGGGAACAAAAGCTGGAG
CTCCACCGCGGTGGCGGCCGCT
CTAGAGTCGACAGCCATATCGCCGCCGCTTTGGCCACCTCCAAACAGCCCCCTCCCCGCA
AAGCCGCACATGCTGCCGGCGGGGCGTCACACACCAGACCAGACCAGACCAGCCCGACAT
TCAACACACACATGGTCTCATGCGGTCTGATGGCTTTCCTAAGCCAACCAGGCCGGCTCC
CAGTGCAGTGACGTGGGCGTGACAGGCCGGGTGCTCCCAGCCGCGTGCCAATTGCCAACC
CCACCCTACGCGAAGGCATTACGCGCCTCACCGTGCATTGCTCCTGCTACAGCCCCTTGC
AACACCGCCGACCTCGGGAAGGTGGAGTTCTCAGCGCGGTGGCCGCTTGCCCCGGCCGGC
AGCTCCGCAGGGCACACGTCACGCGAAGGGCCGCGACGGTTCGAGAACCGACTTGAGGGC
GCCAAACGAGCCCGAGCCGCCGTTGCGCCAGGCGAAACCAGAACCGTAGATTAATGCACT
TGAGCTATTCATTGGAGCGATCTGCCGGGGACAGCGGGTCTGGCGTGCGCGCGATTGGAG
ATCGCAAATTACATATGTCTGCGTGACGGCGGGGAGCTCGCTGAGGCTTGACATGATTGG
TGCGTATGTTTGTATGAAGCTACAGGACTGATTTGGCGGGCTATGAGGGCGGGGGAAGCT
CTGGAAGGGCCGCGATGGGGCGCGCGGCGTCCAGAAGGCGCCATACGGCCCGCTGGCGGC
ACCCATCCGGTATAAAAGCCCGCGACCCCGAACGGTGACCTCCACTTTCAGCGACAAACG
AGCACTTATACATACGCGACTATTCTGCCGCTATACATAACCACTCAGCTAGCGATCCCG
GGCGCGCCAGAAGGAGCGCAGCCAAACCAGGATGATGTTTGATGGGGTATTTGAGCACTT
GCAACCCTTATCCGGAAGCCCCCTGGCCCACAAAGGCTAGGCGCCAATGCAAGCAGTTCG
CATGCAGCCCCTGGAGCGGTGCCCTCCTGATAAACCGGCCAGGGGGCCTATGTTCTTTAC
TTTTTTACAAGAGAAGTCACTCAACATCTTAAAATGGCCAAGCTGACCAGCGCCGTTCCG
GTGCTCACCGCGCGCGACGTCATGGCCATGGCGGCCGTGATCGCCAAGTCGAGCGTGAGC
GCCGCCGTGGCCCGCCCCGCCCGCTCCAGCGTGCGCCCCATGGCCGCCTTGAAGCCCGCC
```

GTGAAGGCCGCGCCCGTGGCCGCGCCCGCCCAAGCCAACCAGCAATTGATGGCCATGCGC
ACCCCGGAGGAGCTGTCCAACCTGATTAAGGATCTGATCGAGCAGTACACTCCCGAGGTC
AAGATGTCGATGGCTCGGGAGGCCGTGATTGCGGAGGTCTCGACCCAGCTGAGCGAAGTC
GTGGGCGTCATCGAGCGCCACCTGGAGCCCACCCTGCTGGCCGTGCACCTGTACGGCTCC
GCCGTGGACGGGGGCCTGAAGCCCCACTCGGACATCGACCTGCTCGTGACCGTGACCGTG
CGCCTGGACGAGACTACTCGCCGGGCTCTCATCAACGACCTGCTGGAAACGAGCGCGTCG
CCTGGCGAGTCGGAGATCCTGCGCGCCGTGGAAGTCACCATCGTCGTGCATGACGACATT
ATCCCCTGGCGCTACCCGGCCAAGCGCGAGCTGCAATTCGGCGAGTGGCAGCGCAACGAC
ATCCTGGCCGGCATCTTCGAGCCCGCGACCATTGACATCGACCTGGCGATCCTCCTGACG
AAGGCCCGCGAGCACTCCGTGGCGCTCGTCGGCCCGGCGGCGGAGGAGCTGTTTGACCCC
GTGCCGGAGCAGGACCTGTTCGAGGCTCTGAACGAGACCCTGACGCTGTGGAACTCCCCT
CCGGATTGGGCCGGCGACGAGCGGAACGTCGTGCTGACCCTGAGCCGCATTTGGTATTCG
GCGGTCACCGGCAAGATCGCCCCCAAGGACGTGGCGGCGGACTGGGCCATGGAGCGGCTG
CCGGCGCAATACCAGCCCGTGATCCTGGAGGCCCGGCAAGCCTACCTCGGGCAGGAGGAG
GACCGCCTGGCGAGCCGGGCGGACCAGCTGGAGGAGTTCGTGCACTACGTCAAGGGCGAG
ATCACGAAGGTCGTGGGCAGTATCTAGACGTCGACCCACTCTAGAGGATCCCC
GCTCCGTGTAAATGGAGGCG**TACGTA**GACAC

III) primers for 3'-RACE

Marker-specific primers:
>CrAadA_F1 Tm=67.7°C
CCGGCGCAATACCAGCCCGTGA
>CrAadA_F2 Tm=69.6°C
CCGGCAAGCCTACCTCGGGCAG
>CrAadA_F3 Tm=66.1°C
GTTCGTGCACTACGTCAAGGGCGA

3' primers
First set:
>Q_T
CCAGTGAGCAGAGTGACGAGGACTCGAGCTCAAGCTTTTTTTTTTTTTTTTT
>Q_O
CCAGTGAGCAGAGTGACG
>Q_I
GAGGACTCGAGCTCAAGC

Second set :
>Q_S
CGAGATCTACACTCTTTCCCTACACTAGACGACGCTCTTCCGATCTTTTTTTTTTTTTTTTTT
>Q_U Tm=63.2°C
CGAGATCTACACTCTTTCCCTACACT
>Q_D Tm=64.4°C
CACTAGACGACGCTCTTCCGATCT

IV) Oligonucleotide used for sequencing:
>CrAadA_F4 Tm=62.1°C
ACTACGTCAAGGGCGAGATCAC

V) Analysis of the FSTs obtained

Shown are the raw unclipped sequences delivered by the sequence provider. The sequence corresponding to the end of the CrAadA cassette is in blue, with the PCR-added sequence or its remnants highlighted in cyan. FSTs are in pink or orange. Poly-A tails are in **bold** and putative polyadenylation signals underlined. Artefacts are shown in red or purple. Unassigned sequences in italics. Note that FST sequencing later revealed that many transformants from transformation #4 (SnaBI cut) carried an uncut PCR product, indicating that the enzyme had not been effective

1) #7.1 control transformant with 3' UTR-containing cassette

PCR2, whole product (major band 1.3 kb, minor at 270)
>#7.1cont_CrAadA_F4 -- unclipped
GGRCTGGCAGTATCTAGACGTCGACCCACTCTAGAGGATCCCCGCTCCGTGTAAATGGAG
GCGCTCGTTGATCTGAGCCTTGCCCCCTGACGAACGGCGGTGGATGGAAGATACTGCTCT
CAAGTGCTGAAGCGGTAGCTTAGCTCCCCGTTTCGTGCTGATCAGTCTTTTTCAACACGT
AAAAAGCGGAGGAGTTTTGCAATTTTGTTGGTTGTAACGATCCTCCGTTGA**AAAAAAAAA**
AAAAAAAAWWKKKKKKKYYYYYYYY*MCCCCAAMYTMYGTTGGKKGTGGGGGGGGWGCSKG*
GMKGGKSSSCYYMYSKKSGSYGKYGRYYGGGYKSGSCCCSGARTGTGTKGAMYGKSCSGC
TGGTCKGGTKWAAACTGTTKTGKGACKYCCCWCMAAYARTATGTMWGGCTTTGCKGGSAG
GGGGCKGTTKSGARGGGGKCAAAGGGGWKMMSATATKRYTGTCCTKTRTACAGCGGCRGC
SAKCGKGGTKGTGTGCCARTTTTTSGTCRGTKKRRAGAGRRMAGYGCTCACGGAGGATCR
SCRAARSYMMTCCRTKGTAMCRSCAGCAWMGRWAMATYAWKCTTGYTRCGKACWCSCYTG
ACSCRMCCCCSTTMCRMAWMGCTGGMGMCACACACACACA

2) #2.1 : VALID, MAPPED

Two FSTs, both of them identified in cut-out PCR3 bands and in the PCR2 product

PCR3, bands cut out from gel
>#2.1A band A (550 nt)
TTCCGAYTGGCAGTATCTAGACGTCGACCCACTCTAGAGGATCCCCGCTCCGTGTAAATG
GAGGCGTARAAACCTAGGCCCGGGCCGGGGGGCCCTTATAACGCCTGCAACACTTATATA
TGCAGCTAGTTTGGTCCCCRAATGGGGGTGCTTATGCTCCTGTGCTCCCAAATGGAATCT
AGTAACGCGGCTAACCTGGCTGGGYCCGCATTCTGGAACCARAGGAACSCACCAAGGAAC
AACCCASTGCCTGGSGTGGYCGCTGCTGGYCWGGCACWCSYAKTACACWTGCACTGSCCG
CCCTGGGCGCAATGCGTATTGTAKGCGTGGACCCGGRCTGTAYYAACTSCCTTCCYCCCW
TACARACCTCAGGKYCCYGCGCATAACMAAAGGCTGT

After the PCR product (recessed by 9nt), there is one nt of unknown origin (R), then Chlamy DNA (Chromosome_16:304054-303727) corresponding to the intergenic region between convergent Cre16.g649800 (PPR3, PPR-cyclin) and Cre16.g649750 (unknown). The poly-A tail is not reached by sequencing.

>#2.1B band B (220 nt)
CAGTRATTGCAGTATCTAGACGTCGACCCACTCTAGAGGATCCCCGCTCCGTGTAAATGG
AGGCGTACCARAACCGTCGCGGCCCTTCGCGTGACGTGTGCCCGGCGGCTCCCGTGTGCG
TGGWATRACTGCGCCTCGCTGTAACGGTGTTGGAGCATCTR**AAAAAAA**

After the PCR product (recessed by 8nt), there are three nt of unknown origin (CAR), then a fragment of the PCR product (401-365 de pALM32, reverse orientation), then Chlamy DNA (Scaffold_19:810984-811035) corresponding to the 3' UTR of Cre19.g757350 (NAC domain, RNA polymerase II general transcription factor BTF3 and related proteins), then a **poly-A tail.**
NB: The position of the polyadenylation site is not one of the 6 already recorded for the recipient gene: the tail starts 4 nt beyond the longest recorded 454 EST. The polyadenylation site may not be the "natural" TGTAA, but rather TGTTG (both underlined).

PCR2, whole product
\>#2.1_CrAadA_F4 -- unclipped
GGRSTGGCAGTATCTAGACGTCGACCCACTCTAGAGGATCCCCGCTCCGTGTAAATGGAG
GCGTACMAAMMYMGKCCCGGSCCKTSGGGYSMTTATWGCSCCGGSRCTCCCGTGTATGTG
GWATGACTGCGYCTCGCTKTAACGGTGTTGGAGCWTCTGARAWMM**AAAAWARAA**WMYMKK
RRSSYSWKSYCCMCAARCTGGGTCCGCATTCTGGAACCAGAGGAACGCACCAAGGAACAA
CCCAGTGCCTGGCGTGGTCGCTGCTGGCCAGGCACTCGCAGTACACATGCACTGGCCGCC
CTGGGCGCAATGCGTATTGTAGGCGTGGACCCGGGMTGCRTCAACTACCTTCCACCCGCA
CAGCCACGAGGGTCAGGCRCATACCGCGAGGCTGTGGGCCGGCCCAAGCAAC**AAAAAAAA**
AAAAAAAAAAAAA*WKSSKKSYKYYYYWMMMMMAAAAAGGAWGYCTKTGGTAGRGGGGACG*
CTRTCTCGGGGGACWCYCTCAKKYKCTYSWTRTSSKGAKCCKGGCACATKKWC

After the PCR product (recessed by 9nt), there are two overlapping sequences. A short section can be attributed to Cre19.g757350 (see above). After a first **poly-A tail**, one can read the intergenic region between convergent Au9.Cre16.g649800 (PPR3, PPR-cyclin) and Au9.Cre16.g649750 (unknown) on Chr16 (see above), followed by a second **poly-A tail**.

3) **#2.2 : VALID, MAPPED**

PCR2, whole product (major band 1.3 kb, minor at 270)
\>#2.2
GTACAKTGTATWYCTAGACGTCGACCCACTCCTAGAGGATCCCCGCTCCGTGTAAATGGA
GGCGTACGTAGACACAAGCAGGAGATCAAGAAGGACGTGCGGCAGGTGGTGGACACGGTG
GAAGCCAGCAGTGCCGGCAAGGGCTACAGCAGCGCAATACGGGTGCCCATCTGGATGCCG
GCCTTCACTCGCCGCCGCGAGGTCTTCATGGGGCGCATCGCCATGGTGGCCTTCGCTGTC
AGCTGCGGTCTGGAGATCTTCACTGCCGACCACCTGGGTCCCATCCGCCAGGTCCAGCTG
TGGAGCGGCCTGGACGAGTCCACTGTGGTGGCCCTCATCATGGGCATCATTGCCTACAAC
GTGCTGGGAGGTCTGGGGCCCTGGTCGCCCACCTTCGCGCCCGAGAACCTGCGGGATGTG
GCCAGGCGGCCGCGCGGCCCGCCCAGCGACCTGCCCGCCAAGGTTGGCGACTGGCTGGGC
ATCAGCGGCTGGGGCTTCACCAAGCGCAACGAGGTGTTCCACGGCCGGCTGGCCATGATG
GGTTTCCTGTTTGCTTTCATCAACGAAATGAAGACCGGCCGCGGCGCACTGGGGCAGGTC
GCTGGCTACCTGGGCATCATCCCCGACGCCGCCTGGTACAGCGCCTGCCTGAACGGCTTC
ATCGTGTTCTCCGCCCTCATGCTGGGAGCCTCCATACTGTTCCCTAGCCGCCTGCAGGGT
TCCAACGCCTCGGAGGATGACATCTACTAAAGCACCGGCGAGCTGTGTGTGCACCAGGGA
GTCGTGCGATGTGTGTCAAGCGGGTCTGCGGTACGGCATATGCCTGCTGCACGATGTGGG
GTGGTGGTAGCTTAGCCGGCGTAGCTGCCGGTGCAACTTGGCTCGGCATGAACCTGCATG
CTGGTATTGGGTAGTGCAGACTCGAGACGGTTGAGGAATGGTAGGTTCAAAATGCGCGCA
AGGACTGTGAGGAGCTGGCATCGGAGGAWGGGGGAAKKTTGGAATTAGCTGTTTGCGCAC
GTGATGKTGGGGTCGGATTGGGGGTTTGCCTGAACCAAATGCACCTCGAGGCYCCGARGA
CGGACYCATCCTGCGCTCTGTCGATATGCCTCACCACCGGTGCCACATGGTTGATTAAAG
GCTAACCGGATSAATSGCTTGGCGATGGCMACAGACTGCCACAACCAAGCGSCCTKTKTK
KGKTTAGACAGTC

After the PCR product (intact), there is Chlamy DNA (Chr_1:8429714-..-8432270) corresponding to exons 3 to 10 (spliced as in the gene model) of the transcript of Cre01.g060850 (PSBS3). The poly-A tail is not reached by sequencing.
NB: There is no 454 or Illumina EST for this gene, so the insertion of the marker may have activated an otherwise silent gene

4) **#2.3 : VALID, MAPPED**

PCR3, band cut out from gel (600 nt)

>#2.3 bande b
CGAAYTTCTAGTATCTAGACGTCGACCCACTCTAGAGGATCCCCGCTCCGTGTAAATGGA
GGCGTACGTAGACACMRCTTTMWTCCCGCCCWKGMTCKATGCACTGGCAAASGTCAACCC
ACRTGTCSCAAGACCTGCATGCCACCTAGCATATTTAATASSTACATGTGGTAATAGCCT
TTGTAGAMRARCSKCCCGYTGRGYKCTG*RATTAWCMCACGGGGYTGCCWWCRCCTCTGSW*
GTTCTTCTGYCRC

After the PCR product (intact), there are 8 unrecognizeable nucleotides, then Chlamy DNA (chromosome_12:589974-589850) corresponding to introns 2 of Cre12.g488050 (FFT5, Fructan fructosyltransferase). The poly-A tail is not reached by sequencing.

5) **#2.4 : VALID, MAPPED**

PCR2, whole product
>#2.4
TTGWCSCGYGTMSRAYYCWMGAMGTYCGAMCCACTTYTCGAAGGAWSCTCGCTCCGTGTW
AATGGAGGCGTWCGTAGACACAACTTTGAACCAATCCTGTAAGGAAACAGTCGCGMWRMA
AAAARRAGGGGGGTCAGCTGCTGTCTGTAATGGAAGGCGTGG**AAAAAAAAAAAAAAAAAA**
AAWSGRARRRMSSKKYYYYKKKGGGGGGGGTGGAGCAGGAGGAGRAGARRAAAAAACAST
TCGMTGTCCCCCCCCGKCYSSSGRKYKKMRSKGSRGRRAAASTGTTTCCCATCCRCCAGG
TCCAKCTRTGGAGCGSCCTGWACGAGTCCMCTGTGRTKGCCCTCATCAYGGGCATCATTG
CCTACAACGWGCTGGKATGTYTGGGGCCCTGRTCCCCCACCTTCGCGCCCWATAACCTGC
KGGAKKTSGSGKKGCGGCCGSGCGKCCCGCTTAGCGACCTGCCCGSCAAGGTTGGCRACG
GGSTGGRCWTCAGCRRCTKGTGCTTCACCAASCGCAACKATCTGTTCTACKGCYGGTTGG
CCAATGAYGGSTTTCCTGTTTSCTTTCRTCAGCAAGRGRGAAYACTGKCCGCCGCSCRYT
GGGGCAGGTCAATAGATGCCTGGKCATCAWCCCCRACGCCKCTWGRTACAGCRCCWGCMT
GAACGGCTTCATCGKGWTCTCCSKCGTCAKGCGGGGAGCWTCCGTACTGTTC

After the PCR product (intact), there is Chlamy repetitive DNA (NonLTR-1_CR). As such, this sequence cannot be mapped unambiguously, but it is possible to read its continuation on the chromatogram below the a poly-A tail:
AACTTTGAACCAATCCTGTAAGGAAACAGTCGCGctgcagtgggagggggtcagctg
Using this additional information, it is possible to map the insertion to Chromosome_14:3326333-3326254, in the 3'-UTR of gene Cre14.g630200 (no predicted function)

Afterwards, a very weak sequence reads Chlamy DNA again (Chr_1:8429714-..-8432270) corresponding to exons 3 to 10 of the transcript of Cre01.g060850 (PSBS3), a probable contaminant from transformant #2.2

6) **#2.5 : VALID, MAPPED**

PCR2, whole product
>#2.5
TGGGCCYGGCSAMWCTMACCTTGAAKCACTTTAGMGGTGTCCGCTTCGTGTYKATGGAGG
CGTACGTAGACACATTCTMCGTGGACACGCCCGAGGCGCTGGAGST**AAAAAAAAAAAAAA**
CTWRRAAAAGTCTTCTTTTGTGGGGGAGRACCCAGMRGGGCASTTGGCCGCTGGCTRTGT
GTCTTTCCCCTGCCTCAWGAAAATGTCCACACGCCGCCSGGACTACWTCCTGGACTGCCT
GGCGCTCCGGCGGCAGCTGGGGCCGGCGCTGGCGCGCGTGTTCGCAGACCCGGCGGTGGT
CAAGGTG**CTCCACGGAAAAAAAAAAAAAAAAAAAAWRRAAAA***MSYYYYYTTTKGGGGRRA*
CCTTCTTCCWGGTGTGCGCCCGGTGGSCYGGGCCTTCTCCTCTCACCCCTRAWRRGGKGK

SCGAGYRGAGAGARRGG

After the PCR product (intact), a short sequence that maps either to Chomosome_12:3268980-3269003 or chlromosome 13:3296909-3296886, then a **poly-A tail**. Afterwards, again Chlamy DNA, unambiguously mapping to Chomosome_12:3269654-3269776, i.e. further downstream the Chr_12 location above, on the same strand. This suggests that we are reading the continuation of the transcript, and that it is spliced. The insertion would be in Cre12.g512250 (protein with HRDC domain), at the start of intron 4, in + orientation

7) #2.6 : VALID, UNMAPPED

PCR2, cut out band (700 nt)

```
>#2.6 700 bp
GTGGACTKGGCGTATCTAGACGTCGACCCACTCTAGAGGATCCCCGCTCCGTGTAAATGG
AGGCGTACGTAGACACTTGGCAGCACTTTCGGCCACTAGTGCAACCTCAACACGGGCGGG
CTGGGGCGGGCACGGCGGACTTGGTGGGGTTATCGGGAGCTGCGAGGCCGGAGGTAGGAG
GCCGCTGAGGGCCACRAATGAGTTGCTAGGCCGCTTGAGGCATGAGTGGAGGCTATTGTC
GGTTTGAGAGATTGGGATTGTCGTTTGGGGCCGTGGCGGTTTGTAACGCTACACGGCAGT
AAGGAGTCAATAACATGGGAATAAGCCCTTGCACGATGACCCGTCTATCGGATACTTATC
AGCACACGGGTGCAGTGGTGCGCATGAACCTGTTGTCCACATGCTGGTCGGTCAACATGT
GTTCCCAAGGCGGATCATTTGTGCGCCTGCACGCTTGTGTGTCGTACGTGACATCGTGAT
TTGGTGACTGGCACCTGGATTTGAATGCGTAACATGGATRAAAAAAAAAAAAAAAAAWGC
AAAACCCKCTCTCTRTTGAGGTTKAATTWARWAATTCSSATGCRTRAAGAAKAAATAATM
TAACCCCAATGWGGT
```

After the PCR product (intact), the sequence hits repetitive Chlamy DNA: first DNA-2-7_CR, then TOC1, ending in a **poly-A tail**. These transposons appear many times on the genome, but never next to one another. This could be an unsequenced locus, or the consequence of a transformation-induced rearrangement.

8) #2.7 : FAILED

PCR2, whole product

```
>#2.7
AAGMGCSYAKCTCCGCKGGWCAGGCAGCACGCAGGTGCCGGTGGYMMGTCCGAGTMCGGC
AGCCTGGACTTCAGCATCACGCAGCTGGACTGTGTGGTCGACCTGGACGGGCACAAGTAC
CGGACCAAGAAGAGCACCACCGCGGCGGGCGCGGCGGCAGCTGCTGCCGCGGCCCCAGCG
CCCAAAAAAAAAAAAAAAAAAAYKSGRAAAAAAACCCCTTTKKGGGGGATMTYGGGGTTG
TGAARAASCTGGCSSCCAGCGGCGAGARSCCSGASCCTGTGCGCACGCACCKGMACGRAT
GATTGTGGTGCCGGASATGATTGSCTCCKTCGYGGGCGTCTACAACKKCAAGACCTTCAA
CCAGGTGGAGATTAAKCCCGAGATGGTCGGCCACTACCTGGCTKAKTTCTYCATCTCCTA
CAARCCCGTGAAGCACTGCCGCCCGGGTATCGGCGCGACTCACMCCMCCCGCRRAATCCC
MCCCACWTAAATGGTCTTCTTGKGSSTCCTGGTTATTTYMGCGGCGCTGWTTTTCTWGGA
TACSCAGCCAGGCCWAAMACGTTYTTTATGTCTGYGCAAGTGCGWCGRCTTCCAATCCTC
GTCG
```

The beginning of the sequence cannot be aligned with the PCR product. The readable sequence (Chromosome_10: 1630531-1630059) starts within exon 7 of Au9.Cre10.g429850, no predicted function. After the **poly-A tail**, the sequence (Chromosome_8: 439197-438932) is very faint is likely to be an artefact. It hits a presumably essential protein, Cre08.g360900 (RPS15, Ribosomal protein S15).

9) **#2.8 : VALID, MAPPED**

PCR2, whole product

>#2.8
GTKWWCMTMWWYWAYTYWMKAMGTYCGACCCACTCTMKAGGWCCCCGCTCCGTGTAAATG
GAGGCGTACGW**AAAAAAAAAAAAAAAAWW***TSKKSKYYYKKYYWMMMMAAAAARMTCCCCC*
CTCCGTGTSSCTGGGGGCGTARGYGGGGSCRMWYCCCCSCCSYWGGKKWGKKKCYCGGGC
*KAAMSYYYKYTKGGGKKYYYCCSCCMCCCCMMMMAAKK*GCGSACAGYTGSWYWATMASCC
ACMMMMMRSTTTGGTGGTCTGGTCGAASCCCCTTGGCCCCTGRMAGTGGATTCCTTGCCT
GCCCGRSGCRGSCTKSSCAGMCMARAAGCTTCTGCGCCACATGCCGCTCCCWCCGCCWGT
GCKGCCGCGGCGGTTATGGTACCTGGTACCTGGTGCGGTGCCKTGGTGCGCGGCACTYCT
GCC**AAAAAAAAAAAAAAAAAAAAAAA***CAAWWYTSMKMGYYTKCWSACCYYARKGGWTYAR*
GRTYYMRYTTTGASTGGCTYKAYTCWRACTATTCCKGTWCATCTSMCTGCCSCCRAYACT
KKTTTGKTTWGSGGMGAGSMGTRMCTWKSMGTKYWACWGSKGGWCGSRTGTSSMTA

After the PCR product (truncated by 5 nt), there is a **polyA-tail**, probably caused by the unwanted **TGTAA** signal found at the very end of the cassette. After 130 nt, you can again read Chlamy DNA (scaffold_27:75383-75179) corresponding to the 5’ UTR of Cre27.g774700 (SGNH_hydrolase), ending in another **poly-A tail**.

10) **#2.9 : VALID, MAPPED**

PCR2, whole product

>#2.9
GTAMTGGMGTATCTAGACGTCGACCCACTCTAGAGGWCCCCGCTCCGTGTAAATGGAGGC
GTACGTAGAGTACGCGACACGGGCTGCCCGGCGTCCAACCCATGACGTGTGAGTGTGTGA
CCCGATTGATTGCTGGAGTAAACACTGTGACCAGGGCTGAAGACGGTACGACTGGCCTTC
CCCTAGACTGGCCGGGTARAGCGGCGCAGTAGCCAACGGCAGTGTCAGCGGATTGTTACC
GAATGGCGGTGAGCATCAGATGTGCGTATTGGGTAAAGCGCTTCTCGGTCTGATCATTGA
TTCTGTCGCGCAAGGGTGTCAATGCTCGAGCCGTCCTCAGCGCGCCCATCATTTGTTCAG
TGCAGGGAACCCAACCCTGACTAAGTTGACATGGTAGTACTGCCGTATTGCCATTCACAT
GAATACGGTCCATGGCCCCAATGCCCCAAGTCATTGCTCTCCTACTGGAGTGGATGAGGA
GCGGGGCGTGACTGGCTCAAATCATTCATCCTCCCGTGCACTCCGTGCTTCCTGTAACTC
AGTACACCT**AAMAAAAAAAAAARRK***SKKRMGYSKKKKTTYYYCWYYAAAAATYCYTGGTT*
KKKGSSSCCSGGTYYMACSGGGGGSCYWWMWMWSSSCCARRKAWMYKKTCSCGAAAAAAA
AAAAASTCGKGGGKTKGGSCCYSMKYYYCYMAYAMMAYAKKKKYWGGGGKTTKGAGKK

After the PCR product (truncated by 3 nt), there is Chlamy DNA (chromosome_9:2341565-2341083) corresponding to the 3’ UTR of Cre09.g400950 (Major Facilitator Superfamily). After that, a **poly-A tail**.

11) **#2.11 : VALID, MAPPED**

PCR2, whole product

>#2.11
TCGGGAYTGGMGTATCTASACGTCGACCCACTCTAGAGGATCCCCGCTCCGTGTAAATGG
AGGCGTACGTAGACACAGGGAGCGTCGGGCCAGGAGCGCGACATGTGGTAAAGGGGAAAG
GCAAAAATTGGAAGGGCAGGGCACTGTGAAAGGGCCGAGGCGCGTCGGCAATAGGAT**AAA**
AAAAAAAAAAAAAAAAAAAAARGRAAA*GGGTTCCCCTTGGGGGGGGGACCCCTCTCGCCT*
TCTGCGCTCAAACCCTCCAATTTTCYYATGACCCCTTGCGGTGAGAAGAAAGGGGAAAAA
YSAAATTAAWAAAYCGSCCGGGKCGCCTTTTAGCCCAATAGTTCCGGAGGAACTGGCSAC
CTACRTATTACSGCGGCSGCGGGGAGGTATTTAGCCTGGGTTTTTTGGCGGGTTSCCGCC
ARGGCGCCSCCCTGTCYRAACGGRACTGGTTTTCCCCCGGMAAAAAATTTTTMCRACCCA

AAGGCTTTTTTTTTCKCGCGGGGTTTCTCKCTAAATTTTCATAYATTGRGAAGAGATTWC
TTCCCGTTGCTCCGCWWMGGAGTTGGGGCCGTGTCTCATCCCAWTGGGGCCAATCACCCT
CCACGTCSGKCTACGMCTCGTTGCCTGGGAGAYCMKTYACCACMCCACTCAACYAAATGC
CSCGCGKCCCTMTCTTWAGTGAYRMCAGACCCSTCTTCCCTCTTTCCTCCRYGMAGMAGA
GAATCTATGCWGTWWTAYCCTCKKTTTCTCTGYGTMTCCMKATYWTACGCGMGKGWAGCW
CACGTGAYWCWCACGYGWCCGCCKCYTCYTTRTTAATCAAKCWTTAAAWRCGTCATCCAG
ACTTRCWGYATGATGCACGWCTCGTCGTTCGTSTGGYRTAGGTWCWYARMKAMWWGATAA
*TCTMGTTTATRATAAAAK*T

After the PCR product (intact), there is Chlamy DNA (chromosome_17:1621018-1620917), intergenic between Cre17.g707950 and Cre17.g708000. After that, a **poly-A tail**.

12) #3.1 : FAILED

PCR2, cut out bands

>#3.1 700 bp band
KACGTCGACCCACTCTAGAGGATCCCCGCTCCGTGTAAATGGAGGCGTACCARAACCGTC
GCGGCCCTTCGCGTGACGTGTGCCCTGCGGAGCTGCCGGCCGGGGCAAGCGGCCACCGCG
CTGARAACTCCACCTTCCCGAGGTCGGCGGTGTTGCAAGGGGCTGTAGCAGGAGCAATGC
ACGGTGAGGCGCGTAR

After the PCR product (cut and filled in), a fragment of the PCR product starting in the HSP70 promoter, but no Chlamydomonas DNA, probably inserted downstream of the transforming DNA at the time of transformation.

>#3.1dTE12BH_premix -- unclipped
GAGMATWWWTGCTGATTCATWYYGCGMACCCGCSCCATCATAGATAGWACYGKACWSCAK
STCCATACACTGCGACRCCGCCAGTGGCATCAGCTCGCGCTAGCATCAACTCCACACTAC
AAAGTCCTTGACGGASCGGCCTGCCTGTGRATTCCMSGACKCACTTGGSGTAGGGTGCCA
TCCTCCTCCCTTTGGGGCACGTCAACAATAACCTCAGCGCASCAAARCARCACGACCAAM
CATGCATGTACTCAACTTTGYCATGACSCGRGTGCKCCASGCCCCCAACCGTCGCTCATA
TATACCCGTAACCCATCCCTGGWGAGTCGGATGCGCTGTGKAAWTACCAKCTTGAYASCA
YGCYGGCCACAAMCATCCATCATCATAAKCGTGSRTG

The PCR producy cannot be read. Instead, the sequence aligns with chromosome_3:1473562:1473840, in 3'-UTR of Cre03.g159300. This is probably due to direct priming on the mRNA, due to partial similarity with the AadA primers used (not shown)

13) #3.2 : VALID, MAPPED

PCR2, whole product
>#3.2
GTGRCTTGGCGTATCTAGACGTCGACCCACTCTAGAGGATCCCCGCTCCGTGTAAATGGA
GGCGTACGWCRACTTCAGCGGCGAAKCCCCTTMGCGGATMCRMMAMRGCTTCCCAGCTGA
GCCCCACCCGAGCTGCCGATGGCCATTGCAAGTGACCATTCATGCATGTATATRTAATGT
TGGCGTCGTGCGCAGCKAGTAKATGAGCCCTCGCCSGACCACCACGATGGKGCGACGCTT
ACTCGCCTGAGGACACAGCCACACCAATTAGACCTTTAGATGTCGGCTGCTTATCTASCT
ATTGAAATAGCAKTGCCTGTCTTCTCRAACACGGACACSCGRGGTCTTAKACGTTGGACC
TGGCCGCTGTGAATAACTCGCGATATTTTASTTGTTATCTGGGCATGTRACTCASAGCTC
AATTATCATACATTGGCCTGGGTGCTTTGCCAGGCGAGAACSGGARAGCGGTCTGGAGAT
TGGGGGCGGTTTCCTTGATGAAGGCCGGAACGTTCGCAACTCGCTTATCATAATCCCTTT
TGGTGTGTCWGCAAAGCTCAACGCACATGCAATTGTGCCTTTATTCATTTATAGGACTGT

GTACGCSACSGCWACGCAGCTGGAACGTTTTTGGCGAGTCGTTCACGCACCTGGATAAAG
TACCGACACAAGCTTCGTTCGTGTATTGAACTCARTGACCGTATTATAACTGGTGTAGCA
AGGTTAGGCAGAGCGGGAAAAAGAGGAGCCTCGCGCCCGCCGGCTTGCTGCGGGAAAATG
GGCCGGAGCAGGAGCAGGAGCCGTGAACGCTCAAAGCGGTCACATCACAGCAGAAGCACG
AGCCGGGACCGAGACCGGTCTGAGCGGGATCGCAGGACCGGTACGTTCTGAGCAGCTTCT
TTAAATGCTGGTTGCATTTGTAAAGCCTTTATGTGA**AAAAAAAAAAAA**C

After the PCR product (cut and filled in), there is Chlamy DNA (chromosome_12:9166015-9166885) corresponding to first intron (in 5'-UTR) of Cre12.g560350 (CNK2, NimA-related protein kinase 2), followed by a **poly-A tail**.

PCR3, cut out bands
>#3.2 band A (1000 nt)
TTTTGACTTGGCGTATCTAGACGTCGACCCACTCTAGAGGATCCCCGCTCCGKGTAAATG
GAGGCGTACGTCRACTTCRGCGGCAAASCCCCTTYSCGGAKACSCGCGGYTTCCCASCTG
ACCCCCMCCCMARTTGCCGATGGCCTTTGCAAG*KCCTGKGTCATGCGGGTATAAATACCC*
GTGAGTCCMARGGYCMGASAGYTTTGCTTGTCTCGYASWGGAWCATCAATAAAGTGTCTC
GAAMCTCAGGTMGGCGSCTGGCAAAGGKTTTCGWGYTAMAGATGRAATKACKTTCCACAT
AAYGCTCAGTGTGTAATCCAWTTTCWTCRYTGTGYCRATGTTCTCSGGGATCGAGTAATC
WKTGGCTCAGTCTTCASTGTKAAAACAACTTACTAACACYAAGTMACWTYAGGTTRTTTT
GTTTTTTCGAYATTCRATTMTCGTAACGGSGCTYGMAATSTCRGGACATWCTCCCKGCWY
GAGACAAAWRAATAKAGCCKAGAKACATGTGTGRAATCGTTACCGAAYCKAGATCTTTAA
WTGTCCCTCASCACTWCAAWCTTAACGSGRCCGGGTAGMAAGCAAGCCCYTCATTATATT
AAGMTMCAG

Sequence of lesser quality, same locus

>#3.2 band B (150-180 nt)
CTAGTWGCTGGCAGTATCTAGACGTCGACCCAYTCTAGAGGATCCCCGYTCCGTGTAAWT
GGAGGCKTACRWMRACTTCASCGGCRAAA**AATGKWMCCTTSYTGGTTYCAAAAAAAAAAS**
YMYKMAAAAAAA*WRATGTSTTTTTGYTCGTCTCTCRTTTTTTTTGGCTCCACTTGGGCTT*
GTATGARGGTGTCCGTCKGTTYGTGWGTTTKAACCGSTGATTTYKWGGTTTGTTYRTTCY
TGGTTSTTGTTTTTTTTTATGGTTTTYYTSTYTYGTYTGYATTTTCTTTWCTTTGAMTMT
TGATWYGGGRWGGT

Contains just the start of the FST noted above, and an early **poly-A tail**.

14) #3.3 : VALID, MAPPED

PCR3, cut out bands
>#3.3 band L (200 nt)
GTATCTAGACGTCGACCCACTCTAGAGCATCCCCGCTCCGTGTRAATGGAGGCGTACTCT
TGGKGTACTGGCATGGARTTCGCTGGCTGTGCATGTGTTTGGTCCTCCTAATGCCCAATC
CCCTCTGTCCCCCTGTTGC**AAAAAAAAAAAAAAAAAAWKAKM**

After the PCR product (cut and filled in), there is Chlamy DNA (chromosome_7:1015855:1015936) corresponding to first intron of Cre07.g319550 (FIST C domain protein), followed by a **poly-A tail**.

>#3.3 band H (350 nt) trimmed
SGGTGGATCTGGCCATGGTGTACGTGTCGTCCGCGTACGASCAGGTGTGTGCATGCKTGT
GTGTGCKTGTGTGTGTGCGTGTGTGCKTTTTTTTTTKTGTGTATGTGAATTGGGCCAAGC
CSCATGGCATCSCGCCCSCCCTCGTATTTCCACCCCGTCGTTGGAACACCATTCCTCCCC
TCCCCATCCGTCCGTCCC**AAAAAAAAAAAAAAAAAR**

Start of sequence (low quality) is trimmed. FST starts within first intron of Cre07.g319550 (chromosome_7:1015996-1016192) and ends a a later position in another **poly-A** tail

15) #3.4 : FAILED

PCR3, cut out band
>#3.4 (220 nt band)
AAGWGWSCGCGMATWATCCTAGCACGTTCGACCCACTCTAGAGGATCCCCGCTTCCGTGT
AAATGGAGGCGTAMW**AAAAAAAAAAAAAAAAAAAAARSRRAA***RRSYYYYYYYKKKKKGGG*
GGGCCCCTGCCTATTTGCATGCGGACCAGASCGGYCTTTYTYCTCTYCTSCCTGGGRCTC
TGCAAAAAGTTGGGGRWKTTTTTTTGTTCTYTTYGGTGYTCYGCCTTTGSTTGYGKGGTT
GGTATTTAGGYRAACTCCWGKTCAMTGGGGSCWCTRAATAKGMMKTTGGAGMSGGWGAAA
TAGCTAAATATAARTTGGCCGTAGCTTTTRATGTTTGAGKCYKTCATATTCTKCRTTTTC
ACACTACATTCTAAGGGTTAGCATSRWCTTTATGYTCTTTTGTWACTCWTGTGYGACYTG
GSAAAAKTAATYGATTATSASAYAYTGKAAGYTYTAAARTYWGKWYMAMTRMWWT

After the PCR product (cut and filled in), there is a single nt before the **poly-A tai**l (on chromatogram, you can see that it is a T)

16) #3.5 : VALID, UNMAPPED

PCR3, cut out band
>#3.5_CrAadA_F4 -- unclipped
GTGRCTGGCAGTATCTAGACGTCGACCCACTCTAGAGGATCCCCGCTCCGTGTAAATGAC
TTTGGTTC**AAAAAAARAAAAAAAAA**MWWWWWKTSRSATKGKYYMMAMMRAMWTCGARCAT
AACCGTGAAACGCCSCAMAGGCCCGTTGGTTGAGGCCTTAAGAATAGCTGCGCCWMRAMM
TGTGTTACAGCACATCAAARCGCATGCCGGTGACTGGATGGTGACCAAATTTCGGGCATT
ATAATCGGGCGACAAGWCRCTCACCTTCTCCAAAGARAATCGACAGGCGGTTTTCAATGG
CCTCATTGGTCAGCTCTGGRARCCRTTGCTGCTGGCGGCGGCGGCCGCCGGCRWRRATCA
ACGCCTCGGCAGCCTGGTGACTAGCCGCGGTGGC**AAAAAAAAAAAAAAAAAA**WYKKWKSS
YSSKYMYMMYYAAMRATGCCACCTTCCCGTGCGAKYTGGAKTCCARSCTGTYKKASCTKC
TGGACGAGGWCWGYWGMSAYCAAAATASAWGRWTCGGSCAACKMAMRGGGASAGGCKGYT
TGCGTAKWGKGCGCWCWTGCGCTTCCYCGCRCACTGAYTYTCWGCGCTCGGTCWTT

The PCR product is truncated. The FST contains at least two polyA tails. Its best (but imperfect) hit is to Chr_7:2564723-2565098 (Au9.Cre07.g332350, unknown function), but the sequence is repeated in genome, at least 12 times: ambiguous mapping

17) #3.6 : VALID, MAPPED

PCR3, cut out band
>#3.6 bande c (200 nt)
TAGRCTTACAGTATCTAGACGTCGACCCACTCTAGAGGATCCCCGCTCCGKGTAAATGGA
GGCGTACGGCGCTGTTACAGCGCACCCWTTAAATTTG**TGTGA**TR**AAAAAAA***CTCSGACA*
TASGSGKGCTGTTTTGCCTGGTGTTCCTTGCTAAACATTGCCCAACATAGTCACTGGAKK
TSGCCSGGGAWWGTCAGCTCCTGSGSATAGT

After the PCR product (cut and filled in), there is an additional G, then Chlamy DNA (Chromosome_3:2440121-2440156), corresponding to intron 6 of Cre03.g166950 (PGM5, phosphoglycerate mutase).

18) #3.7 : FAILED

PCR2, cut out band

>#3.7 band B (1600 bp)
GGGGGGGGTGCATGTGGGCACTGACACAGCCTTGGATGCGGGCGTGTTTGGCAGCATTGC
GGACGGCGGCGTGGGCCTGCTGTGTGGGCTGACGCTTGCGCTGGACATCGCGTGCGGCAT
GAGGCACATCCACAGCCGCAACATCATCCACGGCGACCTCAGCGCCGGGAACGTCCTGCT
CTCGAGCGTCGCTGGCTTCGGATGGGGGCGAGTGGAAGCCCAGGCTAGCGAARTGCKGCC
GTCKGCCSAGAGCRGCRTGRGKGGGGCCGGKGTCTGCRGCACRATTGCGGGTGGGGCTGT
GGATSCGGGTGCACCGGCGGKGGCKGAGAGTGACGCGCAGCTGCTGCGGCCGCTGGCGGG
GCTGTGGCGGCCGCCGGTGACAGCCAAGGTGTCGGACTTCGGGCTCAGTCTCCCCATGGG
CGAGAACCAGACGCACGCCTCAAACCGCTTCCAGGGCACGCCCGGCTATGCGGCGCCCGA
GGTGCTCAGCCGCGGCCGCCTGTCCCTGGCGGCGGACGTGTGGTCCTTTGGTGTGCTGCT
GCTGGAGCTGTGCCACGGCATGCGGTTCAAACACATTCTCGCACGGCAGCAGGCTGCATT
CGGAGGCGTGGCGGGTGCTGGCGTTGCCGGTGGTGGTGAGGAGCGGCCGGGCGACGATGC
GGGCACTGCTGATGGCGGCAGTACCACCGGCAGCGCTCCGGCATGGGCGGTGCTTCCTGC
CACCTGCCCGCCGCAACTGGCGGCGCTGGTGCGGGCGTGCCTCTCCCTCAATCCCTTGGT
GCGGCCATCCTTTGACCAGGTCGTCCGCCAGCTGGTGTCGGTGCTAGCGGAACTTCACGC
CGCCTGCTGTTAGAATCACGTAGGGTGGATTGCTGAATACGGTACAACCCAACTCAACCA
CATTGAATATCTAAGGATGTTGCGTATGGTATCGACTCGCACTTTCCAGCATTGGGCAGG
TGCTCTTCAGTCGTGATGGCAATGTGACTTGGCTGGATGGCGATCAGGCGAATGCRCCCT
ATGGAGAATGGSAGCAATAAGCACGGATTCGTTGKGCGGCTTTGCACCAYCACTTTTCTT
TATTTGCAAGCAAAGRACYAAAAGAGTAGGTGKTTGGGGGCGGCTGTATGATTGGCTGCG
GCATGTTCCACMCYGTTCTGTWAGGCTTGAATGCGCCTTTMCAAACTGAAGGCAGCARGG
GGCAGCCRGGTCMARAAGT

The PCR product is not read. After a bad sequence, there is Chlamy DNA (Chromosome_14: 3746617-3744803, with 6 exons spliced), corresponding to a arge fraction of the transcript of Cre14.g632700 (one of three almost identical protein kinases in tandem at that locus).

19) #3.8 : VALID, UNMAPPED

PCR2, whole product

>#3.8
GCGGGGRYTAGTMGTATCTAGACGTCGACCCACTMKAGAGGATCCCCGCTCCGTGTAAAT
GGAGGCGTACGTAGACACAWCTTTGAACCAATCCTGTAAGGAAACAGTCGCGAAAAAAAA
AAAAAAAAAAAARTGRACTGTCCGCTTGGGGGWAKWTGCAAGCTGGTCGCCCTAGTTCGG
GTTGSYGGTCYCCGAGGAGTTTCGCAWTTTTGYTGGTTGTAACGATCCTCCGTTGAAAAA
AAAAAAAAAAAAAACCAAAAACCGAWGCGGTGGGGGGGTTGAKCTGAGGGATTTCTGMTA
GACAAAGAAACCCACTGCGCTCGCTTTACSTWTACTSTTCTAGATACRGCTYGGACTTAT
ACCTACCMACGGCCGCGGGAGCGAGTTTGTCGTTGCCTTTTTGGCGGATATCGCCAKCRC
TGATCACTGACTAAYYGRWGCTGTTCTTCTCTCCTCACAAAGTTTTACGACCCSAAACGC
TTTTTCTTTCTCGGCGTTGCTCCGTCRRGCTTGCGYCCWTTGGGGAARAATCCCAACAGC
TGCCTCCCSTACGARCTTGGGGCGTGTKTMCTCCCCATTGGGGCCGAACGACCWTCCATG
TCCGTCTRCRCYSGTGCYTTGTGAGGCCATTACTTACCCATMASCAAMTGWCGCGCGGCC
CATCATAAGAGATSCAAAACCATCTYTCMATCTACWTCTYTGCGGAGTGRKAATATATAC
KGTATTATGCTCATGTTGCAWRACGTAWTCCCCTGCTTATRKGAAGGTMACGCYMCGGKT
ACTCAYMCCSKCWCGCGCTMCTCTSAAATWMAAAATCAGWCAATSTACRTTCATCATACG
AGTATGMAYAATCKTCGASTTSTKRGAYWGCAGTCTTCAKMATTAYCTMGAGTCGTMTRM
AAAAAAAA

After the PCR product (uncut !), there is a short 35 nt FST that maps at many locations on Chlamy DNA.

20) #3.11 : VALID, MAPPED

PCR2, cut out bands

```
>#3.11 (500 bp band)
GCGGGGGGSTGCGTATCTAGACGTCGACCCACTCTAGAGGATCCCCGCTCCGTGTAAATG
GAGGCGTACCATTCAGTCACTCCCAAGGAAGCAGGGGGCATGCACGCTTTCGGGCAGCTG
GCATTTGGCGCCCTGCATTGCACCGTCAGAACCCTGGTAACATTGGTGGGTAGTAAACTG
CGAATGTCTTGCAACCAGGTTTTGCCCCCCACCCCCGASCCCCCGACCTCCTACTTCCGC
ACTGCTCACGCTCCATTGCGTCTCTCAACTTTCTTGGGTGTGCGCCTCCCCGCCCTCACA
CTTTTGCCGCCAGGTGACGGGTGTGAAAAAAAAAAAAAAAAAAAWMSRRRRRSSSYYYYY
YWKWTWRRAATCGTGTSCMGWRTGGTSGCCCGTGGTTGAAGGCWAGGGTGACTTTTSCAA
AAGGAGAATCCCAAGGAGCAGGAAGCCKKGGGGAAWTTGGCCCYCCGMGAGGGGGGGAAA
TAATKGGGGTGGAAAAAAAAATATAAWGCCCCCCCCTTSRAAAAACCCCCCCCSTAAAMWA
GGAWTTMCTTCCTCCCCCCCTAAAAGGGGGTGTTCCCCCCYCTCGGCTTGG
```

After the PCR product (cut and filled in), there is Chlamy DNA (chromosome_17:2083314-2083570) corresponding to the 7th intron of Cre17.g712100 (MDAR1, monodehydroascorbate ferredoxin reductase), followed by a **poly-A tail**.

```
>#3.11 (1200 bp band)
GGGGGGGATCGMGTGACTACACGGACGCGGCGGGCGCCAGCCACCAGCTGGATGCCTCGC
TGGTGGTGGTGGGCGTGGGGGCGCGCGCCAACTCCGACCTATTCACCGGCCAGCTGGAGA
TGGCTGCGGGCGGCATCAAGGTCGACCGCATGATGGCCACCAGTGTGCCGGGTGTGTACG
CTGTGGGCGACGTGGCGGCGTTCCCGCTGACCAGCGTGGCCACCGGCCAGGAGTCGCACG
TGCGCCAGGAGCACGTCACGCACTGCCGGTCGAGCGCGGCCCAGGCAGTCAAGGCCATCA
CCAGCACCTCCGCGCCGCCGCCCGCCTACGAGTACCTGCCTTTCTTCTACAGCCGCGTGT
TCAACTTGTCCTGGGTGTTCTACGGCGAGGCCGCCGCGGACGCCACGCCGGTGCACTTCG
GTGACCTGAACGAGGCCAAGGTGTTTGGCTGCCTGTGGCTGGGCGCGGGCGGGCGGCTGG
TGGGCGCCTTCCTGGAGGGCGGCAGCGGCGAGGAGGCGGCCGTGCTCAAGTCGGCGGTGG
CGGCGGGCGTCAAGGGGCTGGACGCGGGGCTGGACACGGCCAGCGGCAGCGGCACCGTGG
CGGCCATCAAGGCCAAGCTGTGAGCTGCTGCGGGGAAGGCGCTGGGGAGAGGTGCTGGGA
CGAGGTGCTGGGAGCATGCGAGGGAGGTGCTTGGAGCATATTCGAGGGAGGTGCTGGGGA
AGATGCTGTGCATTGAGGCTTAGTGGTGGTGACTGGTGGTGGTGGTGGTGAGCTGCAGAG
YTGGCTGTTCGTGCCGTGCGGTGGGGTAGGCAAAGCGTGAACCAGTGCGTGCGCGCGAGG
CACAGCAGTGTRGTGGGGTATGGGGTGTGCATGCATCRGGGCGCTGCATGACAAGAAAAG
GTGGSYTCTCCCTTGCARGGGAAAAGT
```

This is Chlamy DNA (chromosome_17:2083567-2085843), corresponding to the end of the transcript for Cre17.g712100 (MDAR1, monodehydroascorbate ferredoxin reductase), exons spliced together. The junction with the marker cannot be read, suggesting that the cryptic 5' splice site C^GTGGG at pos. 2176 of the transforming DNA was used as a 5' donor, along with the 3' splice site of intron 7

21) #3.12 : VALID, MAPPED

PCR2, whole PCR product

```
>#3.12
GGTGGAGGASWGGAGTATCTAGACGTCRACCCACTCTAGAGGATCCCCGCTCCGTGTAAA
TGGAGGCGTACGTAGACACAWCTTTGAACCAATCCTGTAAGGAAACAGTCKCSAAAAAAA
AAAAAAAAAAAAAAAAAAAAAAAAAAARRRSYYYKKGGGGGGGRSCKYYTTKKKGGGGRAC
CTGSTGKTSTTCTWARATYTTCTCCKTTCTTCGCTGCTATGAAGGTGTATACTGTCTTCT
KCTGCAKCACTTTTCCCAKTTTCCGATGCATTCCGTTGGWKAAGCCGATGGCTTTCACAT
CASACTTAAAAAACCGCCCGCGCTCTYTTTACACCCAATAAAGATGGATAATGGAAGCCA
CCTATTACTGACCCTGCTTGCTCGCACTTAGCCRGCCCTGGCTTTCGGGTAGGATACCGT
CATGACCACTACAKTTAATCTCCGGTCTACTCTTCTCCAACTATTTTACTATCCGAAACG
```

CTTGCCTTCTTCACTCGCGYTGCTTCMTTCCGTCTGACTTGCTTGKGGAAWGGACCAATW
CCCGTGCTGCCTCCAGTAKGGGSCKGGGCCSCGTCTCATTGYGAATGAGGACCATCWACC
TCCGGCTACGGCTACTTATCATGGYSTTGSTTAGCCRATACATCACCAAATASCTMATAC
GCCCMCCGTCCATCGATAACAGATMGCTTATTCCTTCTTTCCTCTGAGAATGATGAGATT
ATWCGATTTTTGCGCTATTAGCATCTGTTTTCACCTGTTTYCCCGYASGTAACCYCAGGT
TACCCYCCCCCTACTCCCCCCTCTGWMACTMAWWAACAATTAARASMARSTTCATCACCC
WWKTATRATCAAACCTCCCTCTTGTGYGGTTATGAGGACKCCGCCGTATCTRCGGTT

After the PCR product (uncut !), there are two superimposed sequences that can be distinguished on the chromatogram, up to the **poly-A** tail :
ATGTGACTRGGTGTGCTGCTGTTGTACGGCTGCCCTGTACGGCCGCAG**AAAAAAA**
aligning to chromosome_10:1630598-1630551, Cre10.g429850 (protein of unknown function conserved in Chlorophyceae)

and:
AACTTTGAACCAAGCCTGTAAGGAAACAG**TCG**CG**AAAAAAAAAAAAA**
a repeated sequence which hits the genome many times

22) #4.1 : VALID, MAPPED

PCR3, cut out bands
>#4.1 (480 nt band)
GCAGTATCTAGACGTCGACCCACTCTAGAGGATCCCCGCTCCGTGTAAATGGAGGCGTAC
GTAGACACGGGTTGCGCGATTTCCGGTTGGTAAGACCGGCGGCTCACCGCAGCAGGTATG
CRAGGTAACTTCAGGTACCATCCGTAAACCGTCGCTTTGCTGCATCGCATACACTGTCTT
TGCGCAACGTTTTCCTTGTGCTCTTTAACACACACACACATATAACTGCTTCATGCTGCW
RMMCTTACAGGTTTGGGR**AAAAAAAGKWYAA**

After the PCR product (uncut !), there is Chlamy DNA (Chromosome_5:377208-377020), intron 6 of Au9.Cre05.g231500 (Zn-finger protein) followed by a **poly-A** tail

>#4.1 (300 nt band)
GCAACGTCGTGGCAGTATCTAGACGTCGACCACTCTAGAGGATCCCGCTCCGTGTAAATG
TGAGGCRTAYGTAGAYAYTGRTTGMGYKMTCTCYGTRTWGGTAAGACCGGCGGCTCACCG
CAGCAAGT**AAAAAAAAAAA**

Based on chromatogram, there are two overlapping sequences after the PCR product, the prominent one can be read as:
GGGTTGCGCTATTTCCGGTTGGTAAGACCGGCGGCTCACCGCAGCAGGT**AAAAAAAAAAA**
which corresponds to the beginning of the 480 nt band, with an early polyadenylation site.

23) #4.2 : VALID, MAPPED

PCR3, cut out band
>#4.2 (750 nt band)
TATCTAGACGTCGACCCACTCTAGAGGATCCCCGCTCCGTGTAAATGGAGGCGTACGTAG
ACACAGACACGTGGATAGCCCAACACRAGACAGGGCTTGACCCATGGAGTTGGGAAGTAG
GGGCGGTGTCGACAGCCRAAGCACCAGGAATAAAGACAGTGRCGGTTGGTGCRAAGCCRA
ATTATTGATTGTGTTGTGACGTGTGCGTGGGGCCGTGGGGGCGTGCAGTCCAGAGTTAAA
AGCC

After the PCR product (uncut !), the FST has no hit on v4 genome, but aligns with other genome assemblies and with the recently released v5 sequence (Jane Grimwood and Mario Stanke, personal communication). This sequence is found at chromosome_8:4,490,830-4,491,010, in + orientation in the 3'UTR of a protein of unknown function (Augustus_11.2|g9033.t1)

24) #4.4 : VALID, MAPPED

PCR2, whole PCR product

```
>#4.4_CrAadA_F4 -- unclipped
GGGRTKAGCAGTATCTAGACGTCRACCCACTCTAKAGGATCCCCGCTCCGTGTWAATGGA
GGCGTACGTASACACTGGGGGAGACTTTCCGTCACGGCCCCRWCCCARCGTCGCTGGCAA
CGTCCACAGMTGTGCRCACCACGCGGCGCTGCTCACTCGCTGCCRACACGACSGCTCCCC
GGCCCTGCCGCGGMCMTGCAGGTGRTCAAGGTGTTTGTAAGCKTATACAGTGACRACTAC
GGCAAGCGAGTGGCCATGGAGAACCTGCAGCGCCTGGAGCCSTRAGTGTCCRCRGGSRCC
GGGGGGCATSKGACGAKGCATCKKGGCGGGGATGGAAARGYSRGGGATGRCATCCSGGWG
CRGGAGGGGTCSAGGAKTGAGGTGSGGCTGCGGGCCCACTTGRWGGMTAGTCTGTGSCMG
CMGMTTGRCGTTTTCAGGGCCGCCACGGCGCGTGTGACGGTCGASGACAGCGGACTCTCG
CCACATCACACCGCRATCTGCTGCAGCTCACATGTAACCGTACCATACACRAAAAAAAAA
AAAAAAAAARRWKKKKSKCYKYYYCACAYAAAACGARKACTTTGAGGACCCARACGAGSG
GCCGCAGGAGTACCCCAACCCCTTTGGCGACCTGTTMAWMRAMRACMRCGAKTACCGGRC
ARTGGCGGKCAAKCGCGTGSAGGAGCGGASGCGTAGCCAGGGCCKCCCGCASCCRCAAGG
YCGCGGGCAKGGGCKGCRTGTGYAAKGGCAGCAGCGGGAGGMKGCGGCGGCTGAGACTGG
TGATGASTAGGTATAATGTCTGTTTGTCAGTGTATACTAACGAGCACGTGCGGGYRCGTG
CAGGAACRGTGGATSGKCYGCWGYATGCAGGTTCATTGATAKCGCAGTGCSACSGGAGCA
CGGRGCCTCMGGCACGCAAGAGCTACTKGWCCTACTGCTAGAGTMCGTCCTMCGGYGCTA
GTCAGATCSCRCCTGGGAATCATCTTCTYGCCTKGTGCGATGGWACGRGGTAAGGGGCAA
GGATGCYGWATYCTGGMATCRYCTCSMCCGGTGCATCTTCYCCCCATTWACGTARCAGTT
KCAYKSKTG
```

After the PCR product (uncut !), there is Chlamy DNA (Chromosome_9:9598215-9596562) corresponding to intron 4 and the end of Cre02.g145000 (Ribosome-binding factor). An early **poly-A** tail obscures reading, but it appears that intron 5 is retained in the chimeric RNA, while intron 6 is spliced out.

25) #4.5 : FAILED

PCR2, whole product

```
>#4.5
GGAYKGGGGAGTATCTAGACGTCRACCACTSTARAGGATCCCCGCTCCGTGTAAATGGAG
GCGYTCGTTGATCTGAGCCTTGCCCCCTGACGAACGGCGGTGGATGGAAGATACTGCTCT
CAAGTGCTGAAGCGGTAGCTTAGCTCCCCGTTTCGTGCTGATCAGTCTTTTTCAACACGT
AAAAAGCGGAGGAGTTTTGCAATTTTGTTGGTTGTAACGATCCTCCGTTGAAAAAAAAAA
AAAAAAAAAAAARRRAAARRRSKSYYYTKKGGGGGGGTCCCATCCGCCAKGTCCAKCTKTG
KAGCGSCCTGGACGAGTCCACTGTGRTGKCCCTCATCATGGGCATCAWTGCCTACAACGT
GCTGGGRGGTCTGGRGCCCTGSTCSCCCACCTKCGCGCCCKATAASCTGCGGGATGTGGC
CAGGCGGCCGCRCKMCCCGCCCAGCGACCTGCCTGCCAAGGTTGGCGACTGGCTGGRCAT
CAGCGGYTGGGGCTTCACCRAGCGCAACGAKGTGTTCCACGGCCGGCWGGCCATGATGGG
TTTCCTGTTTGCTTTCATCSRCSAAATGAAGACCGGGCCSCGGTGCAYTGGGGGYAGGTC
RYTGGMTACCTGGGCATCATCCCCGAKGCCGCCKGGTACAGCGCCTGCCCTGAACWGATG
CATYSTGWAACTCCGCTCTCATGCGGGGAGCCTCCATACTGTTCCCTAGC
```

After the marker, the sequence reads directly, without the PCR-added primers, into the 3'-UTR of RBCS2, up to the end and the **poly-A tail**: this must be PCR contamination from the control

transformants made with the full cassette. Afterwards, a weak sequence from Au9.Cre03.g159300 found in other sequencings, probably a PCR artefact.

26) #4.6 : VALID, UNMAPPED

PCR2, whole product

>#4.6
CTGGCCCCTGGCSRCSCTGCCTTGAMCCACTTTTGWGGYKCTCGCTYCGTGTMKWTGGAG
GCGTMCGCAAAAGCCGGAGGCAACTGCAAAAGCCGCAGCAGCGGCCCAGCAACCCCC**AAA**
AAAAAAAAAAAAAAAAAWTCCTCCTCCTCCKCTTAAAGGGGAAAAAAAAAAAAAAA*GRAA*
ARSKYYYYYYYKKKKKKKGGGGGGMCCCCCCCCCCCCSSGYAAAGAGGRAAAAAAAGGGR
STSSCYYYYCCYCYYMRRRARRRGAAARARRRAAAARAAAWCTTTTGKTGAKGACWGTGC
MTTCACCCCCGGGACTGTATTACATCCGTGGCCCTGYATGRGATTCCGGCACGGGWCGKC
GTTTTMCWTCKTCGYGGCWGSYCCGCCCCTGCCCTTCCMWKACTTAAACARATWMCAGCW
CCTCWTCCTTCCGGGAGWGGGCGTCGGCACTWAGCTGWCCGCTTCSATCWTTAATGCMAR
TAGTTGCYCMTCCKGYATTGCWGKTTCMAGATKGGRTGTTTTGGSTGAYGAGWACTACTT
CCWTATCTGATMGSTATMACATCAGAGCTCYGATGTGGTCAGATTAATGTAAGTGCGATG
GCYTCACCATCGWWGCGGCCWACCTGACCCGTGCCGTTCTCYTAAGTGCAACGCCRTACK
GCCAATGGTWKTGTGWGGCCTTCCCTGCAGSAGCAYCSAACTCCCATTCCTCCTWMTGGC
CTCAGCMTCAGACGACCGATTAGCCTTCWKKTTTASCTGTTGWTTGTTCTTGTCGCTCCC
CWGATAAMCTTSCKCCCTSKCKRCRCKGATTCTCACGCTGCGAAMCYAKMGCGGMRCGGG
TWGCTGGGTTTTRGTTKCTTTCMG

After the PCR product, a Chlamydomonas sequence hitting the genome at several locations, then a **poly-A** tail.

PCR2, cut out band

>#4.6 (280 nt band)
TTCGRCTTGCAGTATCTAGACGTCGACCCACTCTAGAGGWCCCCGCTCCGKGTAAAKGGA
GGCGTACGMAAAAS*CCGGAGGCTCTGCAAACGCCTWTCCCCSWCGCTTAGGAACGGTCGT*
TGGGGGRMTSCTCTGMAAWGTMTGATCAAACWCGGYTGSMAGGKCTTGTCGCCMCCCGRC
TCTGGKSGGCCCCTCCCCTGWGCCTTCCTTGAGGTGSMAGGTGCMACYCCAACTGGATGT
TCCTGMAAGTGGMGMACWTGCTGRCYGATGYCYAASKMKGTGTCCTTASACTAMGGSGGG
GTGGGTGGGGSAGGWCYWTCTKGCGAAGGATGGKGACCACWATACSCGGCRTGKGGGGA
TGAGGRGGGTTCCTATCCTTCKYMTAMACCKAAAAGAAMTGWGGGTCTTCSGCAGCTTGC
TAACGCATAWGATTGRGTGYATTGCMTMGAGRGCYCAGTTKTYGGATCCAGGASAGCCAA
GMAAGAASWATGTAWGCGAAAMAGGTCGCCAMCGGAAGGAATAGSTGSMTGCCCATTAAR
AAWTKAGTTTATGCCCMRMRTTGGCAAAACGCTTTATTWTTCTTTCATACTTAYTMTACC
GMGWWSATAACTCAGAACC

The sequence after the PCR product is poor and yields no hit, either in v4 or v5 or Jane Grimwood's intermediate assemblies

27) #4.8 : VALID, MAPPED

PCR2, whole PCR product

>#4.8
GTWGTWSAMGTMRKWMTYTAGACGTCGACCCACTYTMGAGGATCCCCGCTCCGTGTAAAT
GGAGGCGTACATGCAGATGGATGCGTGCGTGCATTAACGCGTGGAGCGTGGCCGCGTGGA
TCCCATTCCCGCATATGAGCTTACTGGTGCTGCGTGCCTCAACACAATGCAAAAGCATCT
TGCTTGCGGGCGAATGCGCTTATGAATGCGGATGTTACAGAGGGGATAGCGCTTATGCAG
TTGAGATGTATGGTGAAAACAATCGAGGAGATGGAAAGCAAGGTTGCGGCGGAATGTATG
TCAATGCTGTATCTCTCCTAGCATG**AAAAAAAAAAAAAAAAAAAAAAAAAAAAR***GKTKYY*
YSYCWCCCCMYSYAAAAACACGTAYCTCTCCTASSRKGSSSSSSGGGGGSRRRRRWSRSR

CKCCCSSSSGYGGGGRRRYYRSYYSGYGAAAAAAATSGGSGRRWWRRMMTYSCCRTYSKY
AASAASGGSKMGCYWGSGMCSWRAAASKYMCWMMMMARSCGKGGSKYTGY

After the PCR product (SnaBI cut), there is Chlamy DNA (chromosome_7:698506-698252), in the intergenic region between Au9.Cre07.g317300 (MAPKKKK1, Mitogen Activated Protein Kinase Kinase Kinase Kinase 1) and Cre07.g317350 (unknown function). Then a **poly-A tail**

PCR2, cut out band
>#4.8 (480 nt band)
TGAYTGTMGTATCTAGACGTCGACCCACTCTAGAGGATCCCCGCTCCGTGTAAATGGAGG
CGTACATGCAGATGGATGCGTGCGTGCATTAACGCGTGGAGCGTGGCCGCGTGGATCCCA
TTCCCGCATATGAGCTTACTGGTGCTGCGTGCCTCAACACAAGGCGATGAWATCTTGCTW
GCGTGGGTATMATTTTWGGAARTRWWATGTGAAAATTGCGAAAKGTGTTTTTATTTTMTM
WTTTATGTTTCKGSGMCATTTTACGGGCATATKCTMKAGAKAKATTTTTATAMACATTGK
GTTAWTCTCTAWAAAAATATRCTCGKCKTYWCMAWRAAGGGGGAAAGGATTTAWMTGWTA
TCTWAYACTTCMTGRGTWWATYKGAGAGAGTCGTMTCCYCKGGMCMGCTATGGTAAARAK
ATCTTTYCCCYCCCCCCCCCCCCCSSCCMKSTTKMTATCWWTTYTTTCCTTCTCTCTTTC
GTTTTCTATATCMATAATCTATTTCGA

Same, except FST is shorter (chromosome_7:698506-698423) and no poly-A site can be read.

28) #4.9: FAILED

PCR2, whole PCR product
>#4.9
GWGWWCWTGSCKAWTYWMKAMGTYCGACCCACTYWMKAGGATCCCCGCTCCGTGTAAATG
GAGGCGTACG**AAAAAAAAAAAAAAAAAWYTTKKSYYYKRWYCCAYCAAAAA**RGATCCCCC
CYCCKKGKCCCCCCSGGYSYMCGGGGSSYYCCCCCRCSSSCSRGGKKKYYRYTKSSSGSA
AACMWSCRCGCTMATGAAWTTASCATTTWARAKGWTGAMAMYGAMMTTGMTGTTAARYAT
TMSAAAAATATRTTSAARWTWWYAWAMMAKCTSTWAWTYRYRYSTKYKKCRRATYSAAKA
AATGYRMAATAMGAMTGTGYYKCKTTCTCTGSCCAWTCTWGRTCCTTARRGAAACWWWGT
CCAAYASTCWCTCTGGCCKTCGCSCRACARK

After the PCR product, you read directly the **poly-A tail**, with no FST in between

29) #4.10 : VALID, MAPPED

PCR2, whole PCR product
>#4.10
GTWWAYMWMYARKWMTCTAGACGTCSACCCACTCTAGAGGATCCCCGCTCCGTGTAAATG
GAGGCGTA**AAAAAAAAAAAAAAAR**YKTTKRSYYSMSKSYCMWSCMMMCKACGGTCATGSG
YKGGGCCMTGTGTYCCMGTTTCTTGCACAACCACGGGCTACTACGTGACTACSRKSRGCG
GGCAGCAWTTRAGGGTGGGGAGGGAGTGATGGACGTGTGTGCACCWCTGGCCGGGAATSC
RMRGTACTCGCTAGCGATATGCGGATATTGCTCSAARGAAAGCGATTGTGGCCTGAGCGC
TTGCAGCAKAACACGGCTACRAGGTCATATGGCTAGTTTCTTGGGTTGTTTAAATGATCA
ATTCTAAGTGGAGCGACGAGGTCGGGATGTTGCAGTGTCGGAATGCAGTGACGGTTGGCA
CCGTCGCGTGGCCGCATCTGAGCAGTACTTCTAAACRCGTCTTTGGTAGTATGTGGCCTA
GCCTGCCTGTGGGGCACATGAATTGGACACACCGTTCAAGGTTTAAGTTCGAGGAC**AAAA**
AAAAAAAAAAAAMWSKKYKKYSKSRYYYWMKKAYWRKWYCCCCCGKKKGKGGSKYCRGGT
CYWGCASMGGRMYCYCYCCMSMMCCMYWGKGGGKYTTKWARSCCCCMCCWCKCCRCWSYG
YSGTWGMSWSRRRWCCMKYAMMYYYKKSTCMKGGGKKGGRSYMRRMSRCMMCCMMMYYSG

SCSMGGGGKKKGGMYYSRYCCCMCAAMAWCTRGGWSRGGGKKTTGRCCYCWSYWCWMATA
AMWTKGKKWRYYKKKYKKRGKCTKWKWS

After the PCR product, you read directly the **poly-A tail**, then Chlamy DNA (chromosome_7:4591244-4590811), in the 3’ UTR of Au9.Cre07.g346000 (probable adenylate kinase), then a second **poly-A** tail.

30) #11.1 : VALID, MAPPED

PCR2, whole PCR product
>#11.1
TWGGSCCTYGSSAACKMYYCGCTKCGTKCGGTGCCGCGARGCCTCGCAGCCGCAGAACAG
TGGAACCAGGTTYCCTYTTGGTATYCCGTTTGGCTGTAACGGCAGTTGCGGAR**AAAAAAA**
AAAAAAAAAAAAAAMMMMMAAMMMM*YYYYYYYKKKKKKKGGGGGGGRKRMMCCCSSYSYC*
CCCSSSCSSCSSSSSSSGSSSSSCCCCCYCCCCCSCSGRRRRRRGRGGGRRRRRRRRRAA
ARACTWTCCTGCTCTGCTATCGTGGTTCTTGGCTTTGTWCCCTGAACTTGCTYSAATWAT
MGTCCGGCAGCACCGTGCTGCTCCAATATTTCCGTGAGWCAGKRCTTCCGTTTTTTGCGY
TTTTTTCCTCCRGKTGTTTGGCTCACAWRTGCGGTTWCGWTCTTARCKTTTTGCAGCTCS
SSTCCTTCASGCRTGAGTRATTTCCYTTGGAGTCCGCATCGATYTKCCWTTTGRGAGTTG
CGCCCCKGAKTGGSRTCYGGRRGMTGGGCTCTTTTGAGGACGAGGTGKAGKGTCATCTTG
ACCGTTGTTAACGCKKACWGTGAAG

You cannot read the PCR product, probably because the sequencing primer is too close to the AatII site. After position 46, the sequence that can be read on the chromatogram is:
......GCAGCGCAGACAGTGGACCAGGTTCCGATGGTATCCGTTGGCTGTAACGGCAGTTGCGGAGA
This corresponds to chromosome_16:2391524-2391584, intergenic region upstream of Cre16.g666300, followed by a **poly-A tai**l.

31) #11.3 : FAILED

PCR2, whole PCR product
>#11.3
GTTGGGKAMGCTGCGGCGGGCGGACAGCTGGAGG**AAAAAAAAAAAAAAAAAAAAWYSRAAA**
AMSSYYYYYYKGGGGGGRAACTKYGCGGAAAAGAA*KCGCGTTATSTAGACTCCTGTTGGC*
CCCCCTCTTGTSCGGCCCGCCGCGTCGTTTGCACAGCTMASAGCCGCTTTCCCCACGGGT
TCTCCCCGGTATCTAAACCCATCASGSCYWCGTGMTYGGACTTCGGTGGGGGTCTTTGCT
CTRGGCTTTCTGTTTGTTCGGGTTTTGKTGGTTTATCCGAAGAAKTTTTTTTCATCACAA
TTAAGGAGCCCCGCTGGCTCTGTTTMCCCCGATRAACTCCGGACACTTCTCGGCTGTGGC
GRATCTTGARGGTTGCCGTGAYGTTKTTCTTCTGTTTTTTGGGGTTGATATAGACGTTGC
GGCTGTTGTASTTGTACTCAYGTTTGTGCTCAGMATGTTTCTTTCATCACTGAAGTCTAT
TCTTCACTCATCGAGGCTTTTTAWGTCGGAGTTCCCTTCATGGTGAAACATTCCCCACGG
CTTGCTCTGGMAGAAGTTGGGGAMGAAYATCGTGCGCTGCTGAACATATCTCTCTTTCAT
GGACGACATGATCTGTCGAGGGCTTCRTGTMCCACWAGYTATGTSTGACGCGGCGCGCGC
ATWAGAAATGASAGACCCGAYGTTGTTTCCTSTGCCCSGCAMGCTGASCACMGAGTTAGA
TGAGTTAATAKCTGTTTTGWSAATGTGGCTATCSGCTGGWCGCMCGGAYCCMTGAACACT
GGCCGTTTATTCTCAACAATTYAAWAGRAGGCCAYCACCCTGWKACWGAYCYCCTCCTTG
TCACGWCGRCGGYACCTGAGCTGTCTCTAGTTCKGTMTCAMARCCTMRCTGCAGCGCCCT
CTTTAAGGKCRGCTGACSTCAAGCCCTCTGGTCCCCACCTCCTACCTCTGCGCATCCTMT
CACGCCTGCTGTGCT

No hit on genome, with whole sequence or with the part before the **poly-A tail**.

32) #11.4 : VALID, MAPPED

PCR2, whole PCR product
>#11.4
TGGGMAGGGGCATWYCTAGTGCTTGTGAATGTGGCAACCAGCTCACCGAGGTCTCTCGGG
CAAGCCTGGAGATTGCGGCGGGCGTGGTGGCGGGCGGCGCCGCAGGCGGCATGTTGTCGC
CCAACGCTGCACGTGCCTTTGGTGGCGCCGGCACGGGCCCTGGCAGCGTGGCGAGTGGCG
GCGCGCCTGGAAGCGGAGGCGCCAAGCTGCCTGGCGTGGTGCCCACGTGGCGGCAGGTGG
CCAGCAACCAGCTGTACGACGAGCTGTATGACGGGGATCAAGCAGGCGTGGCGGAGGGCG
ACGAAGGTGCTGGAGCGGTTGAGGGGCAGGCCGACGGGGAAGGGGCGGCAGGCGTGACGT
ATAGCAACGTGATATTTGGAGAGGGCGAGCAGGAGGTCGGAGACGGCGAGGAGGAAGAGG
ACGCTTGAGGTTAAACAATTTTGCATTCGGGCGTGCGAAGAGGTTTGGAGGCAGTCTACT
ACGCCAGAGGCTTTGTGTTTTGCTGRCAGAACAGGCGGGGGCTTGAGAATTTTGCGGACC
GAAGTGCGAAGAAGACACTCAGACGGTGACCAGGAAAATGCATATCGTATAGGCGATGTG
ACTGTGGCCGTGACTGTGGMCARTAMCGTATGCWGTGATTCGRCAAGCAKGGCGTGTTCT
CACCCCRTAKGTACGCCSTTTGGCGCGGTGCCAAAGACSATTGCAGATCAKACGCCAATA
CGGTRGTCCCTMSCCCCGCCGTTRTCTGTTTCGACRCSSGTTGTGATMCCCTAGATCGAG
GTGTTGAGAAACACYRAAYTAKGGAGGTCCCATYCWTCYRKCKCARATGTCAACTCSTKM
WGGGAKCTKRATCSAGYMYTWCCACAACCCACAGTATTTAGGGGRGGGWCCMTYCCKGKC
SKCGGYGGSTGTSWASSKSGKWSAGGGACACACACWCMCCYCACSGSCGCCGSMGSCGGG
GAAGAGMSGGCCTCTTCTCKCCRCMRMRKAGGTGGGGGGAKAGARAGAAAGAAA

This corresponds to chromosome_4:704462-705281 in the last exon of Au9.Cre04.g215800 (no functional annotation). Before that, all that can be read on the chromatogram is GCAGTAATCT, which corresponds to the end of the PCR product, ending two nt before the AatII site used for the transformation.

33) #11.5 : FAILED

PCR2, cutout band
>#11.5 (400 bp band)
ATAGCCACCGGGGACGCGATAAAACCACTATCTTTTTAGCGGGATTYTGAYCGAATAACG
CCACGRTWWTKTWKTCCTTTTAAAAKGRCKATCCTTTTKYATCCTGGGGTTGTTTGAATT
TTCCCCCATWGTAYYTTTASKTCGAAAACAYCGCTGGGCGCTAAAGTGTCTCTGTAATTG
ATCTSTTCTTCCSCCCACTCGGGATTTATCCCCTCTTATCCKCCTA

No similarity to the PCR product, no match on genome

34) #14.1 : VALID, MAPPED

PCR2, whole PCR product
>#14.1
GGRGYMGGCGTATCTAKACGTCGACCMCTCWASAGGWCCCSGCTCCGTGTAAAKGGAGGC
KTACGYARAYMCRRMRKGYRSRYYSGARCTGGGAASCAAAAAGGSMAGGSRGSRSKKGGA
CAWSTKKGSMGACMGYGSRAMKTACTTMWASCCGWGGYKGARCTCTTGCTAGGCTWCCCK
CCKSWGACTGGYCCSRWTGGGMCACATAAACTTCTWGGCCASGWMGGAKGCGGARAAGGA
SCRCRAACAGGGCCASTCCGACTRCMKCKCCKKCCWWRSYASSATGCGCMTMGCCCRGGY
TCGGYTCCGRATSMMMTSARKCCGCYTGSYWYYTTAYGTGTTTTSWAWGTCCSTSTGCCC
CGAWCTGGTCMMAAWCTGAASCMCTAACTKCRMCAYAAAWGAMGGARCTGGCGRCWKGTG
TGKTTTGTCRWGRCCSGCGGGTGCSYSAKAWCCSGAATGTGTMACCGCTGARTTCSACGA
ARATG**AAAAAAAAAAAA***TTGTTCSTTTGGYKMCKYAYACACTTACAAAGGATCTCCACMA*
RCRATCKGTGGCAGSRCCMGCTTCTGTGGCTYAAGCTTGTTACGGACTCSSTTGAAGARM
YCYCTTTCYGGAKCWKCTTGAGCGGCTGTGCTTYWSSRGRAAKCGGYCTGGSAGTACKGA
RMGYCACTGTYRCGAACSGSGATGGSCYGCTGCCYGGAARGTTWGACTGYGKSTTTGKTC

*TCKYTGKGTRWWKTCGGSCCTGCG*GGTTATTGATRACATGCMMCAAKKGGAGGAGGATGY
CAAMMMTACGCCAAGTGCGTCCTGGAATCCACAGGCAGGCCGCTCCGTCAAGGACTTTGT
AGTGTGGAGTTGATGCWARMRRCGARCTGATGCCAMCTGGCGGCGTCGCAGTGTATGGAG
CTGCTGTACAGTACTATCATGTTGGGCGCGGGTTGCGCARAGTTGAARTCACCAMTAAAG
GTGCTCRCTTTTGASKACAGTGCTTACGAAAGTAGCGTCCGCAMGKGTCCGCATTKGCTA
GGCATGGAACCAAAGTGCACTCCGGCCACAMMACCYCMAGKGTGGAAGTTGCCSYTTGGG
GGWCRCRTTGACATACCAWGGAATTCCKTGGATKCCKGTAKAATCCCATATACSAATCWC
GCAATCTGG

On the chromatogram, after the PCR product, you can read below an early polyA-tail the sequence :
GGCTTGTGGGCCGGAGCTGGGCTCAACGCGGGCAGGGAGGAGTTAGACATGTGGGACGA
CCGTGTGAATTACTTCAAGCCGAGGCTGACACTCTTGCTGCAGCATACCCTCCGCCGACT
GGCACGGCTCAGAGTTTAAACTTCTAGGCCAGGCGGACCCTGCGGAGCGCGAACAGGGC
Which maps to Chromosome_14:2909515-2909394, intron 7 of Cre14g.627600 (Dynein heavy chain). After that, another sequence from the same locus, but further downstream (Chromosome_14:2908783-2908718). The **poly-A tail** read further downstream is that of another gene, Cre18.g749500 (60S ribosomal protein L28), probably a PCR artefact, as is the fragment of the PCR product found downstream

35) #14.2 : VALID, UNMAPPED

PCR2, cutout bands
>#14.2 (800 bp band)
AGACGTCGACCCACTCTAGAGGATCCCCGCTCCGTGTAAATGGAGGCGTACGTAGACACT
TTCGGCCACGTGGAAATGGTGATCATTACCCTGCCTGACGGCACCACCGTCAAGGCCGCA
CGCAAGACCCTGCACGCGTGTGAGACCAAGGGCGCTGTGGCTGCACTACGCCGCATACTG
AACCAGGAGCTGGCAGGGCTGGCTGCTGCCGCGGGCTGCGAGCACGCGGTGCAGTGCCTG
GGCTACCGGCTGCCGACTGATGACGACGAGCGGGCGGAGCTGCTGCTGAGCTGTGCGGAG
GGCGGCTCCGTAGAGAACCTGCTGAATGCGCTGGCCAAGGAGCACTTTGATCGCATTGAC
AACGCGCCCGAGCGCCGGCGCACCGGCAAGAACAAGGACAAGTATGAGATGCTGCCGTAC
CCCGGCAACACGCTCATGGATGAGGCTGACCTGAAGGGCATGCTGCGKGCCWTG

After the PCR product, the sequence aligns perfectly well onto Chromosome_12 a number of times, with splicing (2 or 3 exons depending on the genes): 4837773-4838273, 4851541-4852133, 4854996-4855146, 4869726-4870319, 4871001-4871526, 4874404-4874550, 4905266-4908803, 4925725-4926318, 4929186-4929336, 4944276-4944869. This corresponds to a series of almost identical kinase genes specific of Chlamydomonas, of which three show an equally good match to the FST.

>#14.2 (320 bp band)
GTTGGGAYTGCGTATCTAGACGTCGACCCACTCTAGAGGATCCCCGCTCCGTGTAAATGG
AGGCGTACGTAGACACTTTCGGCCACGTGGAAATGGTGATCATTACCCTGCCTGACGGCA
CCACCGTCAAGGCCGCACGCAAGACCCTGCACGCGTGTGA**AAAAAAAAAAAAAAAAAAA**
AAAA*TTCACCGGGTCTTCTTCCCCTTTGGGCGKTACRCYTGAWTTTWCTGGTTCTTCTGC*
GCACAAAACTTTCCCTTTCTCCTAACCRCTTGAAGTGGGGCGGGAGGGAATTTCATMTYA
YATTTAAAACCCCSGCYGCGCTCKTTTTACCCCCAAWTTCTCSASACAACTCTCGCCCCC
TWTATTATTACCGCCTGCTGCTGGGTAGTTATTCGTGGTGTTTTTGTGGGTRACCACCRT
GGCGCCSCYCTCTTYTACTGYCACTTGTCTTCTTCTKTAAACACAGATTTATAACACSCA
AGCCTTCTCYTCTCTCRCGSGTGGTTCCTCCAGASACTTTCYATSATGGAAATAATMCAC
TGCTGCTCCCCCAGGAGAAGTGCGGGCGTCTCACTCRCACTGTGTSYGATCAWCATCTCT
CTYAGGCTACGACACATTGCCKTGCTGATGCGWTACYAYACCACYAACTARATGTRCGCG
GSSCGATCATSAAGTRATAGYCARAGCMGWCTYCTYTYTTTMTCTWGCAGGATGARGACC
TAGTCRKTATTGCTMCGRTTKCTRGCTTAKCTMGAYTTCACGGMWGGTAGGCCASSTWCY
CRTWCCYAAYCRCCSTACTAATCAATCAACTGTAATACGTMGYCMGAATCGWSATRTSAG
TCSCTGCATTGTCWMGTMKTMGGCYCTCYSMAWYAKWAATCTGMGGT

Here, the transcript starts similarly, but ends in a **poly-A tail**

36) #14.3 : VALID, MAPPED

PCR2, cutout band

>#14.3 250bp_CrAadA_F3 -- unclipped
TCGTAGTCGTGGCAGTATCTAGACGTCGACCCACTCTAGAGGATCCCCGCTCCGTGTAAA
TGGAGGCGTACGTAGACACAACTTTGAACCAATCCTGTAAGGAAACAGTCGCGCTGCART
GGGAGGGGGGTCAGCTGCTGTCTGTAATGGAAGGCGTGG**AAAAAAAAAAAAAAAAAAAAM**
SAAAAAR*SSSYYYYYKWWWAARRGGCATYSSYCCYCTGGGGGGGAWCCTCCSWTGACGAR*
TTTAAATTAAAAYACCTGGAACCCTCTTCACCGGCAYCSKCCKTCTCGTTGCMTACARGA
GGCTGCTTTTGTTTACTGTTWKCMCMTCMCGGAGGTTAAACTRGTGGCCMGCGGTCCCTA
ATTTACASCCAACTTAATGTTACAGCGACKTTTGTCTATTTCGTATACTAAGGRAKTTRW
CCTATTCTTTKTTTCTATTTCCAGCSAAATTATTTTTTTTCCGATCATTTTMCAWYTATT
TTASGATTGTSGCATCWAATGTAATMT

After the PCR product, you read a sequence found on chromosome_14:3326333-3326254, downstream of and convergent with Cre14.g630200 (a gene conserved in Volvox, Micromonas, possibly Paramecium), followed by a **poly-A tail**.

37) #14.4: FAILED

PCR2, whole PCR products

>#14.4
TGGAWCWGGMRTWATCTAGACGTCGACCCACTCTTAGAGGATCCCCGCTCCGTGTAAATG
GAGGCGTACGGT**AAAAAAAAAAAAAAAAAAAAWSGRAAAAR***SSCCCCTTTTATTTTAGCC*
TGTYTGCTCCCYACACGTTCGCGCCTCRKCGTCGGTTCCACACCAGACAGCCCTCTTCCC
CGGGGGGGAACCTCCAYATCTCTRCYTTTTTTCCTCTKCAGGGGGAASTCCACTCTCCTC
TTGTACACTSAARCCTCCCAKTTTCCAATGATWCCTCGCCCCTGACCCGGGAGATTTGGC
TCASACAAAAAAAAAAAASAGCWCGGTGGGGGGCTCGATGTTGAGACRCKCTYGCCACCT
ACATATTACCGCGKGTGGKGGCACRTATTTMSCCGTGGTTTTCTGGCCGGGTACCGTCRA
SGCSCCGCCCTGTTCRAACGGCACTTGTTCTTCCCCGGMAACAKAKTTTTACSACCCRAC
GCCTTCTTCACTCACGCGGCGTTGCTCCGTCAGACTTTCGTCCATTGCGRAASATTCCCT
ACTGCTGCCTCCCGTARGAGTTTGGGCCGTGTCTCCSTCCCRATGTGGYCSATCACCCTC
TCWGGTCRGCTACGCATCGYTKCSTTGGTGAGTCRTTACCCCACCAACTARCTSATGCGC
CGCGCGTCCATCTRTAARTGACARCMSAAKCCKTCTTTCCTTTTTCCTCCATGCGGAGGA
AAAAACTATCCKGTATTAKCCYCKGTTTCCCGGCGTTMTCMCGATCTGACAGGYAGGTTG
CSTAYGTGTTACTCACCCGYCCSCCGCTACCTTTTAAAARCAAGCTATWAAAARGTCCAT
MMTACWTGYATGTATCAAGYMCCCGCCTTGTTCGSCTTGYMRSSTTYWCTMMKAAWATAA
TAAAM

After the PCR product (recessed by 7 nt), a 2 bp FST, then a **poly-A tail**.
